# Genetic Regulation of *Caenorhabditis elegans* Lysosome Related Organelle Function

**DOI:** 10.1371/journal.pgen.1003908

**Published:** 2013-10-24

**Authors:** Alexander A. Soukas, Christopher E. Carr, Gary Ruvkun

**Affiliations:** 1 Center for Human Genetic Research, Massachusetts General Hospital, Boston, Massachusetts, United States of America; 2 Department of Medicine, Harvard Medical School, Boston, Massachusetts, United States of America; 3 Department of Molecular Biology, Massachusetts General Hospital, Boston, Massachusetts, United States of America; 4 Department of Earth and Interplanetary Sciences, Massachusetts Institute of Technology, Cambridge, Massachusetts, United States of America; 5 Department of Genetics, Harvard Medical School, Boston, Massachusetts, United States of America; Stanford University Medical Center, United States of America

## Abstract

Lysosomes are membrane-bound organelles that contain acid hydrolases that degrade cellular proteins, lipids, nucleic acids, and oligosaccharides, and are important for cellular maintenance and protection against age-related decline. Lysosome related organelles (LROs) are specialized lysosomes found in organisms from humans to worms, and share many of the features of classic lysosomes. Defective LROs are associated with human immune disorders and neurological disease. *Caenorhabditis elegans* LROs are the site of concentration of vital dyes such as Nile red as well as age-associated autofluorescence. Even though certain short-lived mutants have high LRO Nile red and high autofluorescence, and other long-lived mutants have low LRO Nile red and low autofluorescence, these two biologies are distinct. We identified a genetic pathway that modulates aging-related LRO phenotypes via serotonin signaling and the gene *kat-1*, which encodes a mitochondrial ketothiolase. Regulation of LRO phenotypes by serotonin and *kat-1* in turn depend on the proton-coupled, transmembrane transporter SKAT-1. *skat-1* loss of function mutations strongly suppress the high LRO Nile red accumulation phenotype of *kat-1* mutation. Using a systems approach, we further analyzed the role of 571 genes in LRO biology. These results highlight a gene network that modulates LRO biology in a manner dependent upon the conserved protein kinase TOR complex 2. The results implicate new genetic pathways involved in LRO biology, aging related physiology, and potentially human diseases of the LRO.

## Author Summary

Lysosome related organelles (LROs) are specialized, membrane-bound organelles that share many common features of canonical lysosomes. Mutations in critical components of LRO biogenesis lead to human diseases of immunity, blood clotting, and pigmentation. In *Caenorhabditis elegans*, LROs are the site of accumulation of aging-related autofluorescence and the vital dye Nile red when fed to living *C. elegans*. Through classical genetics we show that the LRO is regulated by a conserved genetic pathway involving serotonin, a mitochondrial ketothiolase, and a proton-coupled solute transporter. Though previously thought to be linked in an obligatory manner, through systems level analysis we show that accumulation of *C. elegans* LRO Nile red and autofluorescence are mechanistically distinct processes. Contrary to the prior notion that LRO Nile red indicates lipid stores, we show that LRO Nile red is not correlated with, and may be anticorrelated with, *C. elegans* lipid stores. Using hundreds of candidate gene inactivations that disrupt Nile red accumulation, we determined which LRO regulatory genes specifically interact with 6 genetic mutants known to have altered LRO biology, identifying changes specifically dependent upon target of rapamycin complex 2 signaling. These data reveal relationships between LRO biology and aging and metabolism in *C. elegans*.

## Introduction

Lysosomes are ubiquitous, dynamic, membrane-bound organelles that serve a major degradative role within cells. Lysosomes receive material through the endocytic pathway and are its terminal compartment [1]–[3]. Lysosomes also receive material via the secretory pathway and directly from the cytoplasm [1]–[5]. Proteins, lipids, nucleic acids, and oligosaccharides received from endocytic, secretory, and cytoplasmic compartments are degraded within lysosomes, and breakdown products are exported to cellular anabolic or catabolic processes [4]–[6]. Lysosomes function in diverse cellular processes including cell surface receptor turnover, destruction of pathogens, antigen processing, digestion, starvation responses, tissue remodeling, ion storage, autophagy, aging, and plasma membrane repair [2], [3], [7].

Lysosome related organelles (LRO), also known as gut granules in *Caenorhabditis elegans* [8], are a heterogeneous group of specialized, membrane-bound cellular compartments that share many of the features of classical lysosomes [3], [9]. Specifically, they have acidic pH, contain acid hydrolases, and lack mannose-6-phosphate receptors [4], [9]. LROs subserve a specialized set of functions such as the production and storage of pigment (melanosomes), immune defense (neutrophil azurophilic granules), and blood clotting (platelet dense granules) [9]. Human disorders of LRO biogenesis or function are characterized by defects in pigmentation (melanosome), immunodeficiency (neutrophil), bleeding diathesis (platelet), and neurological disease [3]. In *Drosophila*, defects in LRO biogenesis or function lead to altered eye color, caused by defects in the trafficking of pigment granules [3], [9].

In *C. elegans*, lysosome-related organelles are the site of microscopic autofluorescence which accumulates as animals age [10]. LROs are easily recognized in the worm by their autofluorescence and birefringence under polarized microscopy, and mutations that disrupt LRO function also disrupt age-dependent accumulation of autofluorescence [8]. *C. elegans* LROs also serve as a cellular reservoir for zinc, preventing toxicity of high dietary zinc [11].

*C. elegans* LROs are the site of accumulation of the vital dyes Nile red and BODIPY-labeled fatty acids when these substances are fed to living *C. elegans* with *E. coli* as a nutrient source [8], [12]–[14]. This, together with the lipophilic properties of Nile red and BODIPY-labeled fatty acids, led to the erroneous conclusion these dyes reveal the storage of neutral lipids and that the Nile red stores are the site of fat storage in *C. elegans* [15]–[19]. By a number of microscopic and cell-biological techniques, LROs do not co-localize with *C. elegans* neutral lipid droplets [14], [20]–[23] and mutants defective in LRO formation do not show alterations in neutral lipid stores or lipid droplet staining [12], [13]. Finally, additional vital dyes that highlight the LRO, such as Neutral red, TRITC-dextran, and acridine orange, accumulate in an identical cellular compartment as the acidified compartment marker Lysotracker Red [8], [12]–[14].

Here we use the LRO uptake of Nile red in *C. elegans* to probe for genes that regulate the biology of the LRO. Serotonin decreases Nile red accumulation in *C. elegans* [17]. Forward genetic screening for mutants that continue to accumulate Nile red in the presence of serotonin revealed serotonin independent uptake and storage of Nile red in many, independently isolated mitochondrial ketothiolase *kat-1* mutants [18]. We further identified *skat-1* (for *s*uppressor of *kat-1*), as acting downstream of serotonin and *kat-1* to positively regulate LRO Nile red. *skat-1* encodes a predicted 9-transmembrane domain protein orthologous to proton-coupled, vacuolar, amino acid transporters. Without functional SKAT-1, the high LRO Nile red phenotype caused by *kat-1* mutation is fully suppressed. Because the LRO also is the site of intestinal autofluorescence, we determined whether *kat-1*, serotonin, and *skat-1* also regulate LRO autofluorescence in a parallel manner to LRO Nile red accumulation. Surprisingly, we found that while blue wavelength LRO autofluorescence was decreased in *skat-1* mutants, similar to LRO Nile red, green wavelength LRO autofluorescence was paradoxically increased in *skat-1* mutants. These observations suggest that *skat-1* regulates essential LRO functions and possibly through modulation of LRO pH.

To identify other regulators of LRO function, we surveyed by RNAi a set of 407 genes that have been reported to affect *C. elegans* Nile red [16] along with an additional 164 genes annotated to modulate *C. elegans* metabolism. Seventy-nine of these gene inactivations significantly altered LRO Nile red in wild type animals, as measured by quantitative microscopy. These 79 gene inactivations were also tested in six other mutant genetic backgrounds known to have altered LRO Nile red and intestinal autofluorescence in order to illuminate the genetic architecture underlying LRO biology. The results demonstrate distinct genetic pathways converging on regulation of LRO Nile red and autofluorescence.

## Results

### Serotonin negatively regulates accumulation of Nile red in *C. elegans* lysosome related organelles

We used the *C. elegans* feeding Nile red assay to highlight the lysosome related organelle [12], [14], [20], [22]. The lysosome related organelle is also the site of autofluorescence accumulation during aging, and this autofluorescence is thought to represent an age pigment, or lipofuscin [8], [13]. We hereafter refer to the feeding Nile red assay as LRO Nile red.

**Figure 1:**
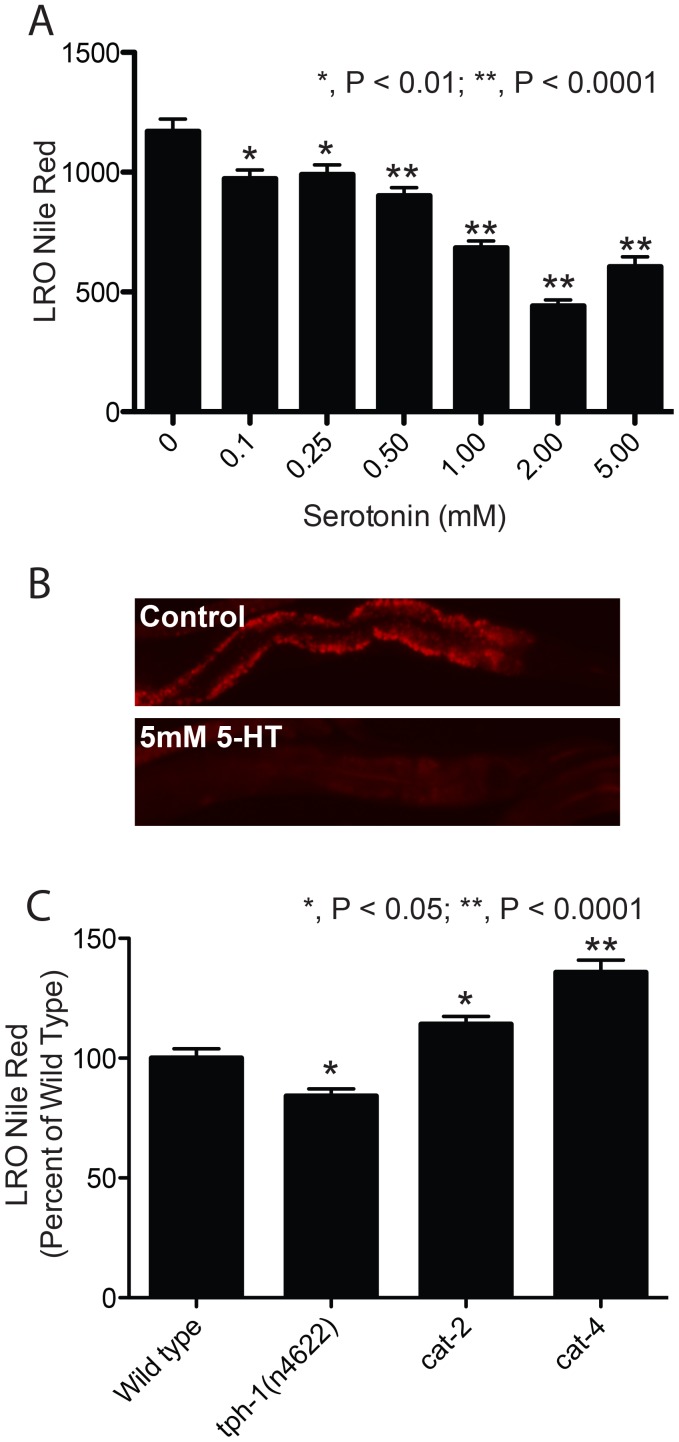
Exogenous serotonin reduces LRO Nile red. (A) Exogenous serotonin suppresses LRO Nile red in a dose dependent manner. Animals were treated for 48 hours with doses indicated. (B) A representative image of an animal treated with 5 mM serotonin shows near complete absence of LRO Nile red. (C) Mutants lacking serotonin do not have a large effect on LRO Nile red. A null mutant in *tph-1* (tryptophan hydroxylase) causes a modest decrease in LRO Nile red whereas a null *cat-4* mutant (GTP-cyclohydrolase) which lacks dopamine and serotonin has a small but significant increase in LRO Nile red. *cat-2* mutants have defective dopamine production but preserve normal serotonin synthesis. (N>25; significance by ANOVA with Bonferroni correction.)

Serotonin is a tryptophan-derived biogenic amine that serves as a signaling molecule in metazoans and subserves a variety of behaviors including feeding and satiety from nematodes to mammals [24]–[27]. Because serotonin is synthesized in *C. elegans* ciliated neurons [28] and because ciliary neuron mutants such as *tub-1* and *bbs-1* show altered Nile red accumulation in the LRO [18], we tested whether changes in serotonergic tone alter LRO Nile red. We added serotonin to wild-type *C. elegans* and observed a dose-dependent decrease in LRO Nile red (Figure 1, A and B). LRO Nile red accumulation was less dramatically affected by mutations that induce either states of high and low serotonin. In mutants carrying null mutations in the serotonin synthesis genes and therefore lacking serotonin, a modest decrease in LRO Nile red was observed (Figure 1C). Mutants in tryptophan hydroxylase *tph-1* show a decrease in LRO Nile red whereas mutants in *cat-4* (GTP cyclohydrolase I) which have defective serotonin and dopamine synthesis show an increase (Figure 1C). To control for the effects of loss of dopamine, we analyzed *cat-2* mutants, which lack dopamine but maintain serotonin, and their levels of LRO Nile red were marginally higher than wild type. Thus elevated serotonin causes a decrease in LRO Nile red, and there was no clear effect of decreased serotonin levels on LRO Nile red.

### The mitochondrial ketothiolase *kat-1* functions in a genetic pathway with serotonin regulating LRO Nile red

**Figure 2:**
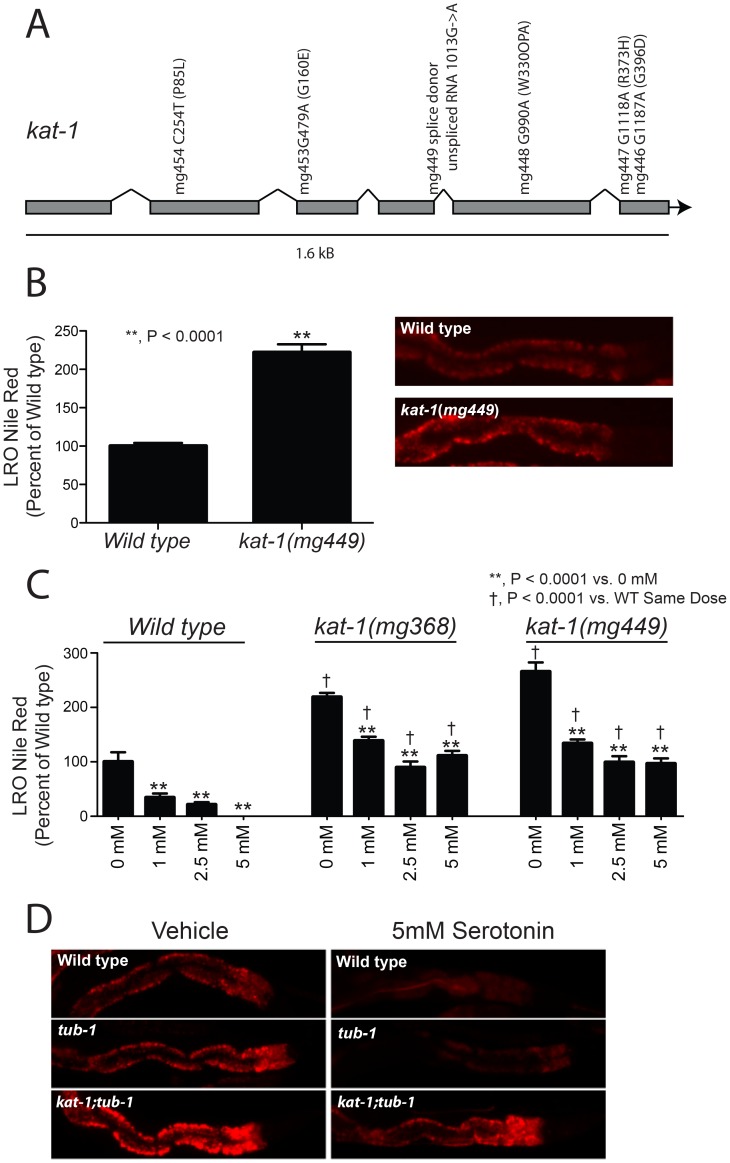
Mutations in the ketothiolase *kat-1* lead to resistance to exogenous serotonin treatment. (A) Six mutations in *kat-1* were identified in a forward genetic screen for resistance to the Nile red accumulation reduction induced by exogenous serotonin. Two mutations (*mg449* and *mg448*) led to predicted truncated proteins and therefore the mutations are predicted strong loss of functions. Nucleotide numbers refer to the spliced RNA of *kat-1* except for *mg449* where the unspliced *kat-1* RNA is used as a reference to indicate the loss of a splice donor site. (B) In the absence of exogenous serotonin, *kat-1* mutants show a more than doubling of LRO Nile red on day 1 of adulthood. (N>25, significance by unpaired, equal variance Student's t-test.) (C) *kat-1* mutants show attenuated loss of LRO Nile red in response to 55 hours of exogenous serotonin treatment. Wild type worms lose essentially all LRO Nile red, and while *kat-1* mutants lose approximately the same quantitative amount of LRO Nile red, they preserve 50% of their LRO Nile red level. (N>25, significance by ANOVA with Bonferroni correction.) (D) Wild type and *tub-1* mutants have full response to serotonin, reducing LRO Nile red to low levels. On the other hand, the *kat-1;tub-1* double mutant, like the *kat-1* single mutant, has significant resistance to exogenous serotonin treatment.

To determine effectors of the *C. elegans* LRO response to serotonin, we conducted a genetic screen for resistance of LRO Nile red reduction by exogenous serotonin treatment. Forward genetic screening for high LRO Nile red fluorescence in living animals in the presence of a level of exogenous serotonin that causes a decrease LRO Nile red in wild type animals yielded ∼100 high LRO Nile red mutant strains. We mapped the mutants harboring the brightest residual LRO Nile red signal and identified 6 alleles of the mitochondrial ketothiolase *kat-1* (Figure 2A). *kat-1* mutations elevate LRO Nile red in strains that also carry a mutation in the *C. elegans* homologue of mammalian tubby gene, *tub-1* [18]. *kat-1* mutations were also identified in a genetic screen for elevated intestinal autofluorescence [29].

LRO Nile red in *kat-1* mutants without serotonin treatment is more than twice that of wild-type animals at day 1 of adulthood (Figure 2B). When treated with serotonin, wild-type animals lose virtually all LRO Nile red over 48–72 hours. However, serotonin- treated strains bearing either of two independent *kat-1* alleles show retention of approximately half of their starting levels of LRO Nile red (Figure 2C), although the absolute decrease in LRO Nile red is similar between wild type and *kat-1* mutants. *tub-1* mutant animals maintain sensitivity to exogenous serotonin and show decreased LRO Nile red upon serotonin treatment (Figure 2D). As in the case of the *kat-1* single mutant, *kat-1;tub-1* double mutants retain high LRO Nile red even when treated with serotonin (Figure 2D). Thus in a *kat-1* mutant, Nile red uptake into the LRO is partially resistant to exogenous serotonin. But the kat-1 mutation induced increase in Nile red in the LRO remains responsive to a *tub-1* mediated output of the ciliated neurons, because null mutations in *tub-1* strongly enhance the LRO Nile red storage phenotype of many *kat-1* alleles [18].

**Figure 3:**
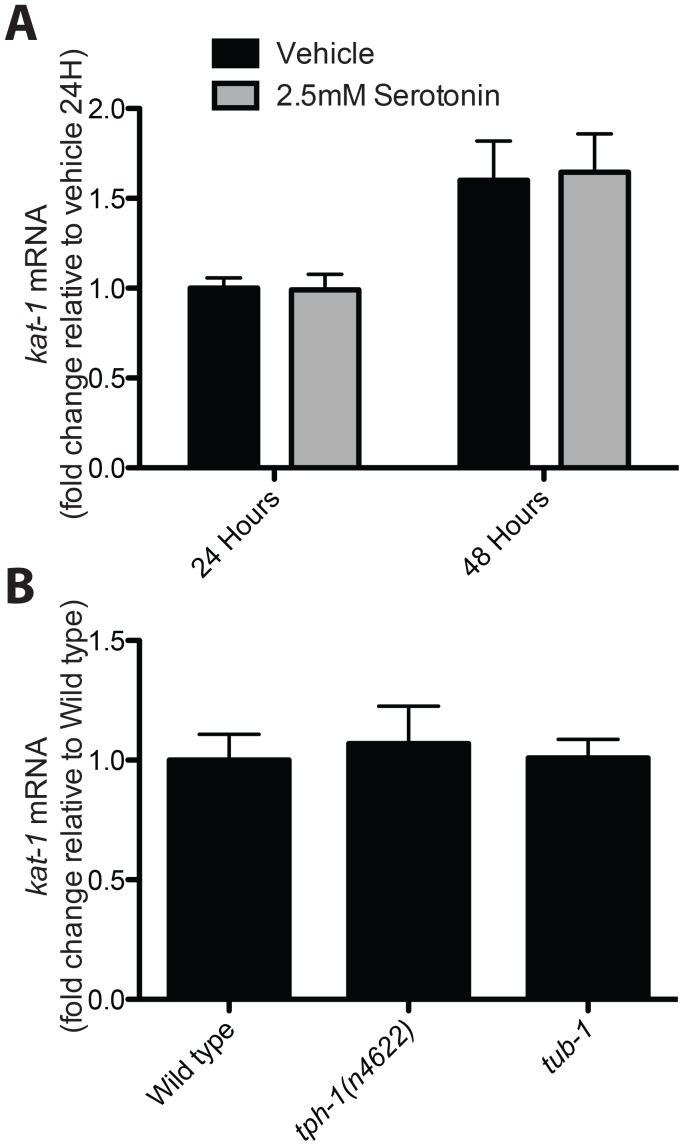
*kat-1* mRNA is not transcriptionally regulated by serotonin, and *tub-1* regulates LRO Nile red in parallel with serotonin and *kat-1*. (A) *kat*-1 mRNA abundance does not change with exogenous serotonin treatment. (B) *kat-1* mRNA abundance is identical in *tph-1* and *tub-1* mutants. (A and B, N = 3; significance by ANOVA with Bonferroni correction.)

We investigated the means by which the serotonin response depends on the KAT-1 thiolase. *kat-1* mRNA levels after treatment with exogenous serotonin did not change (Figure 3A). *kat-1* mRNA was also unchanged in both *tph-1* mutants and *tub-1* ciliary mutants (Figure 3B). Thus *kat-1* is not transcriptionally regulated by serotonin.

### High LRO Nile red of *kat-1* mutants is suppressed by loss of function in the 9-transmembrane domain transporter *skat-1*

To determine which of our ∼100 mutants with altered LRO Nile red might be in a pathway with *kat-1* and serotonin, we inactivated *kat-1* by RNAi in each of the newly isolated mutants. The majority of mutants showed an additive increase in LRO Nile red when *kat-1* was inactivated. However, one mutant with slightly low LRO Nile red in a *kat-1(+)* background showed a near disappearance of LRO Nile red when *kat-1* was inactivated by RNAi. We mapped this mutation and identified a missense mutation in the annotated transporter F59B2.2, which we named *s*uppressor of *kat-1* or *skat-1*.

*skat-1* encodes a predicted 9-transmembrane domain protein orthologous to yeast and mammalian proton-coupled amino acid transporters (Figure S1, A and B). As *skat-1* is predicted to be in an operon with two upstream genes *F59B2.5*, and *F59B2.3*, we constructed a GFP promoter fusion to the most upstream gene in the operon, *F59B2.5* to determine the site of expression of *skat-1*. This revealed expression in head, tail, body and ventral nerve cord neurons, muscles of the vulva, and intestine (Figure S1C).

**Figure 4:**
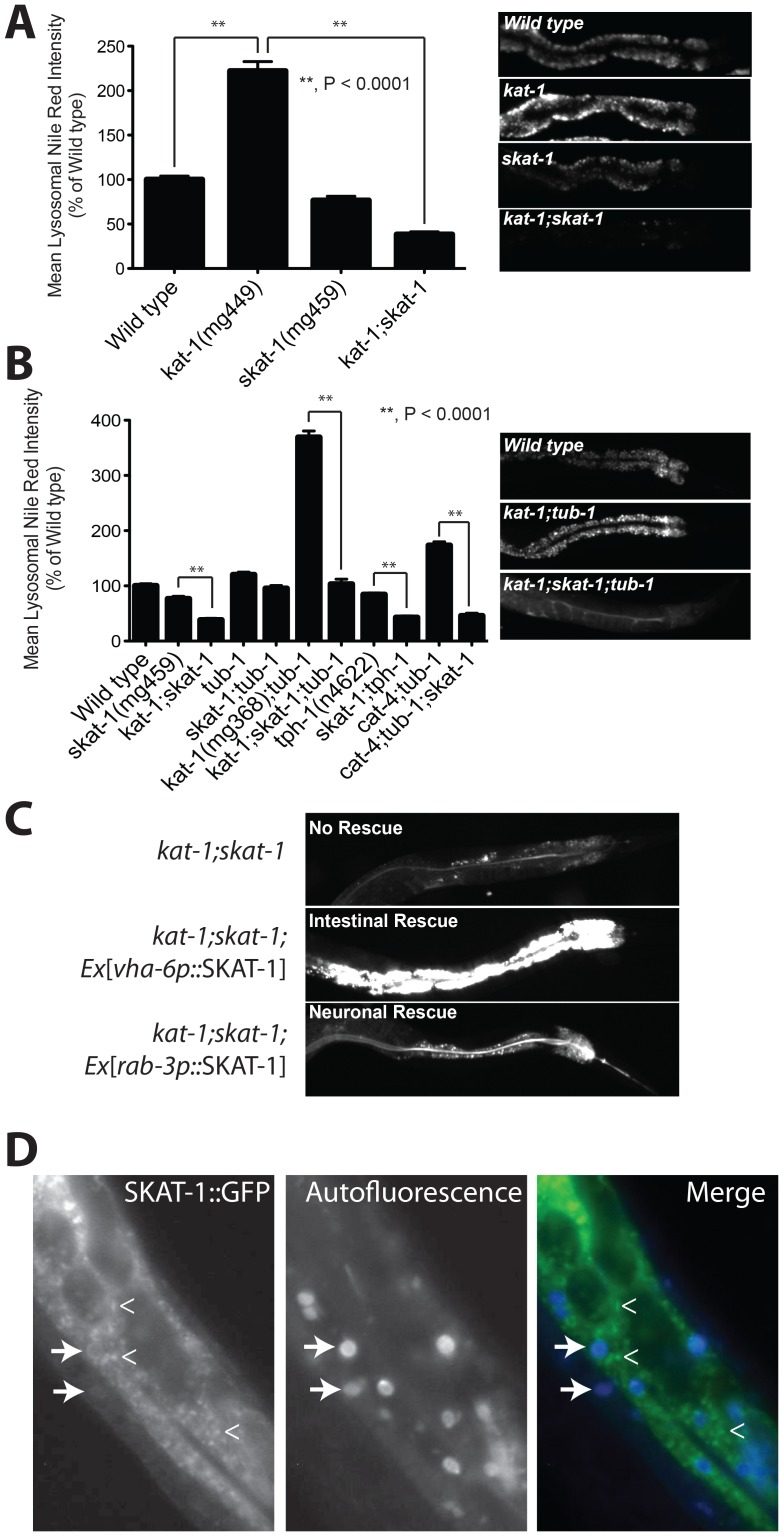
Suppression of the high LRO Nile red phenotype of *kat-1* by mutations in the proton coupled amino acid transmembrane transporter *skat-1*. (A) a *kat-1* mutant has a doubling of LRO Nile red. A *skat-1* single mutant has a 30% reduction in LRO Nile red. The *kat-1;skat-1* double mutant show a near complete absence of LRO Nile red, indicating that *skat-1* completely suppresses the high LRO Nile red of a *kat-1* mutant. Representative images of individual animals are shown in the right panel. (B) As with the *kat-1;skat-1* double mutant, loss of *skat-1* in mutants lacking serotonin (*tph-1* and *cat-4*) show a synergistic decrease in LRO Nile red. The dramatic increase in LRO Nile red in the *kat-1;tub-1* double mutant is strongly suppressed in the *kat-1;skat-1;tub-1* triple mutant (representative image shown at right). (A and B, N>25, significance by ANOVA with Bonferroni correction.) (C) *skat-1* acts in the intestine in a cell autonomous manner to regulate LRO Nile red. Expression of *skat-1* under the intestine specific promoter *vha-6* in a *kat-1;skat-1* double mutant fully restores the high LRO Nile red phenotype of a *kat-1* mutant, whereas expression in the nervous system under the *rab-3* promoter does not. (D) SKAT-1::GFP expressed under the intestine-specific vha-6 promoter highlights hollow vesicles which are also positive for autofluorescent material, likely representing lysosome-related organelles (*arrows*). A large amount of SKAT-1::GFP is localized to smaller, more punctate cytoplasmic structures and does not co-localize with autofluorescent material (*angle brackets*).

While *skat-1* single mutants show a non-significant trend towards decreased LRO Nile red, double *kat-1;skat-1* mutants manifest nearly absent LRO Nile red, far lower than *skat-1* single mutants (Figure 4A). Given that *skat-1* could potently suppress the large increase in LRO Nile red in *kat-1* mutants, and since serotonin and *kat-1* are in a genetic pathway with each other, we also hypothesized *skat-1* might interact with serotonin pathway mutants. In *tub-1* mutants, loss of *skat-1* has a small, nonsignificant effect in LRO Nile red (Figure 4B). However, in *kat-1;skat-1* mutants, *kat-1; skat-1;tub-1* mutants, *skat-1;tph-1* serotonin-deficient mutants or *cat-4;skat-1;tub-1* serotonin deficient mutants, LRO Nile red was reduced 3–4 fold, and microscopically to levels much less than wild type (Figure 4B). All that was visible in *kat-1;skat-1;tub-1* triple mutants was a Nile red streak in the intestinal lumen, with essentially no detectable LRO Nile red (Figure 4B, *right*). This indicates either in animals lacking serotonin or *kat-1* that *skat-1* synergistically reduces LRO Nile red, and provides further evidence that *kat-1* and serotonin lie in a genetic pathway regulating lysosome-related organelles.

We used expression information to guide construction of tissue-specific *skat-1* rescue constructs. As we observed both neuronal and intestinal expression with the *F59B2.5p*::GFP transgenic strain, we attempted intestinal rescue of *skat-1* with a *vha-6* intestine-specific promoter and rescue in the nervous system using a *rab-3* pan-neuronal promoter. We injected SKAT-1::GFP fusion rescue constructs into *kat-1;skat-1* double mutants in order to see re-animation of intestinal LRO Nile red in rescued animals. Only with intestinal rescue constructs did we observe an elevation in LRO Nile red, indicating that similar to *kat-1* [18], *skat-1* regulates lysosome related organelles in the intestine in a cell autonomous manner (Figure 4C). Based upon prediction algorithms, SKAT-1 is predicted to be cytoplasmic and localized either in the plasma membrane or in a membrane-bound structure (Psort II, http://psort.hgc.jp/). SKAT-1::GFP was visualized in spherical, autofluorescent, cytoplasmic gut granules (Figure 4D), but more brightly in smaller cytoplasmic structures that did not co-localize with intestinal autofluorescence. These structures were abundant and excluded from the nucleus (Figure 4D). These data suggest that SKAT-1 at least partially localizes to the LRO and might directly regulate LRO physiology.

### Mutation of *skat-1* regulates LRO accumulation of age pigment and vital dyes

**Figure 5:**
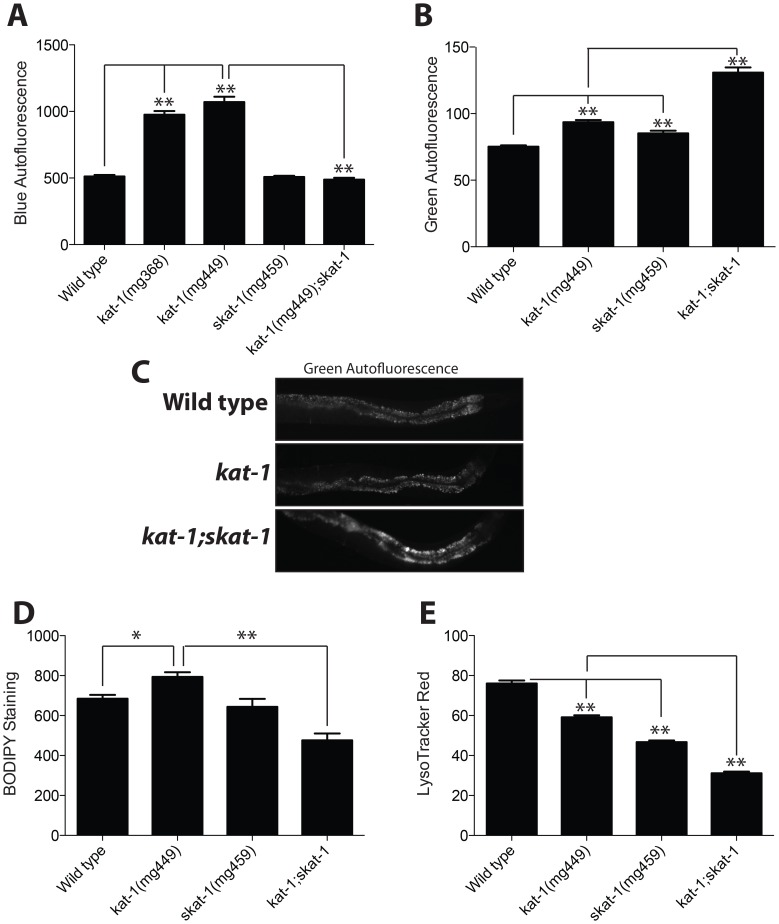
LRO autofluorescence and vital dye accumulation are regulated by *skat-1*. (A) A *kat-1* mutant has high blue spectrum autofluorescence, which is concordant with the high LRO Nile red phenotype. Mutation of *skat-1* in *kat-1* mutants fully suppresses accumulation of LRO blue spectrum autofluorescence with age. *kat-1*(*mg368*) and *kat-1*(*mg449*) show similar phenotypes in blue spectrum autofluorescence. (B) *kat-1* and *skat-1* both show elevated green spectrum autofluorescence that is synergistic in the *kat-1;skat-1* double mutant. (C) Green autofluorescence in a representative day 1 adult animal for each genotype. (D) C1-BODIPY-C12, which is accumulated in the LRO, is also increased in *kat-1* mutants and fully suppressed like Nile red in the *kat-1;skat-1* double mutant. (E) In contrast, LysoTracker Red is decreased in *kat-1* and *skat-1* single mutants and synthetically decreased in the *kat-1;skat-1* double mutant. (N>25; *, P<0.05; **, P<0.0001 by one-way ANOVA with Bonferroni correction.)

The lysosome-related organelle is not only the principle site of feeding-Nile red accumulation, but also the site of autofluorescent material that may represent age pigment or lipofuscin accumulation [8], [13]. To determine if the accumulation of age pigment in the LRO is also regulated by *kat-1* and *skat-1*, we examined intestinal autofluorescence in these mutants. Intestinal autofluorescence accumulates in wild type *C. elegans* with age, and in progeric strains of *C. elegans* such as *daf-16* and *rict-1*, intestinal autofluorescence is elevated [10], [30]. Intestinal autofluorescent material emits fluorescence in both blue and green wavelengths. We examined the effects of *kat-1* and *skat-1* mutations on both spectra. *kat-1* mutants have elevated autofluorescence in the blue spectrum, and much like the Nile red synergistic phenotype, *kat-1;skat-1* double mutants show full suppression of the increased blue fluorescence in the *kat-1* mutant (Figure 5A). Both the *kat-1* loss of function allele isolated in this study (*mg449*) and the reference allele previously identified (*mg368*) show quantitatively similar increases in LRO Nile red and blue spectrum autofluorescence (Figure 2C and Figure 5A, respectively). Conversely, *skat-1* mutants had elevated green spectrum intestinal autofluorescence, and this was synthetically exaggerated in double *kat-1*;*skat-1* mutants (Figure 5, B and C). This analysis indicated that *skat-1* synergistically increases intestinal green-spectrum autofluorescence while reducing the accumulation of blue spectrum autofluorescence and Nile red in that compartment.

To determine the physiological handling of diverse vital dyes by the LRO in *kat-1* mutants, we examined accumulation of the labeled fatty acid C1-BODIPY-C12 and the Vital dye LysoTracker Red. C1-BODIPY-C12 accumulates similarly to Nile red and blue spectrum autofluorescence in a *kat-1* mutant, and the C1-BODIPY-C12 accumulation is fully suppressed in a *kat-1;skat-1* double mutant (Figure 5D). In contrast, accumulation of the vital dye LysoTracker Red in the LRO [13] is reduced in *kat-1* and *skat-1* mutants and is additive in the *kat-1;skat-1* double mutant. Thus, *kat-1* and *skat-1* do not globally increase the uptake of vital dyes in a non-specific manner into the LRO.

In order to confirm that Nile red, LysoTracker Red, and autofluorescence accumulate in the LRO, we analyzed PGP-2::GFP transgenic animals fed Nile red as a vital dye and conducted imaging for blue spectrum autofluorescence, PGP-2::GFP, and Nile red (Figure S2A). These data confirm that Nile red and autofluorescence perfectly overlap with PGP-2::GFP in the LRO (Figure S2A) [8], [12]–[14]. We also analyzed PGP-2 overlap with LysoTracker Red, finding that while all LysoTracker Red positive granules also contain PGP-2, not all PGP-2 positive granules contain LysoTracker Red (Figure S2, B and C).

Autofluorescence accumulates with age. Confirming that the animals being compared were at an equivalent age, we found egg to egg time for the following strains to be within 1–2 hours of 70 hours at 20°C: wild type N2, *kat-1*(*mg368*), *kat-1*(*mg447*), *kat-1*(*mg449*), *skat-1*(*mg459*), *skat-1*;*tub-1*(*nr2004*), *kat-1(mg368*);*skat-1*(*mg449*);*tub-1*(*nr2004*), and *kat-1*(*mg449*);*skat-1*(*mg459*). At 72 hours after egg lay, all strains examined had laid eggs and adults had 6–8 corporal eggs and oocytes, a further confirmation of equivalent developmental timing into early adulthood. Thus, *skat-1* decouples the LRO from Nile red import induced for example by a defect in KAT-1 ketothiolase activity, reducing blue autofluorescence while more strongly inducing the accumulation of green autofluorescence.

### Systems level analysis of LRO autofluorescence, LRO Nile red accumulation, and fat storage

**Figure 6:**
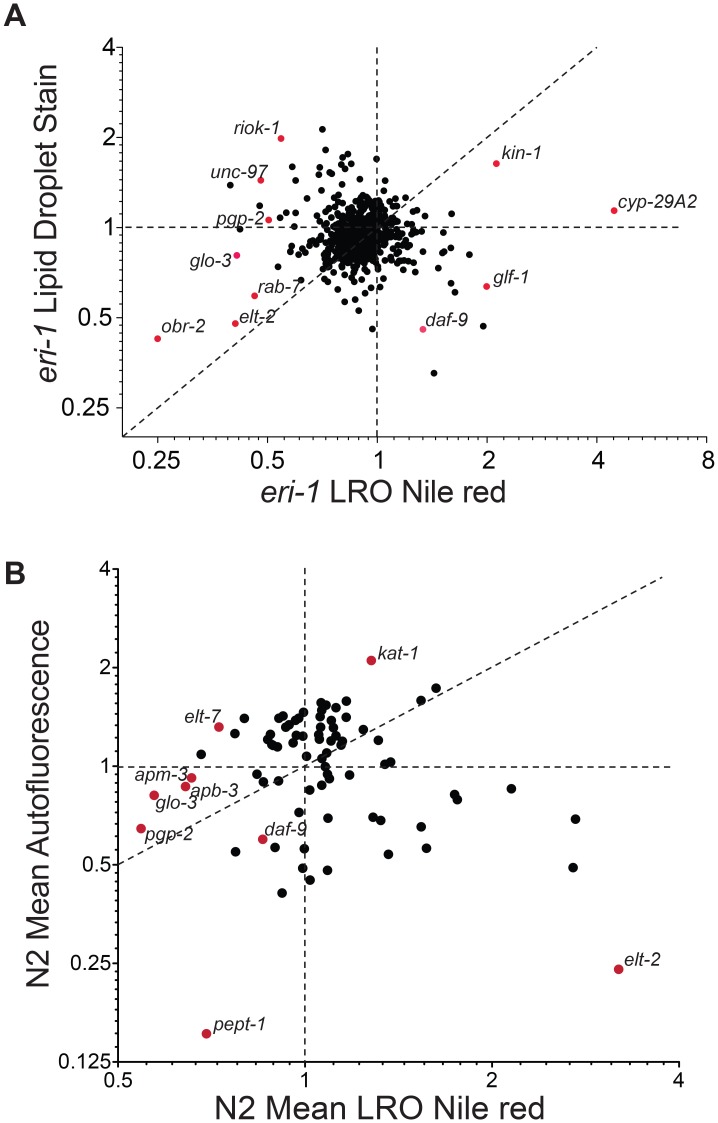
LRO Nile red is not equivalent to and most often discordant with blue autofluorescence and fat mass. (A) LRO Nile red versus fixation-based lipid staining for 571 gene inactivations in *eri-1* enhanced RNAi mutant worms show that LRO Nile red is not equivalent to lipid mass. There are a limited number of gene inactivations that show concordant changes in LRO Nile red and lipid mass as determined by fixation staining, however, many gene inactivations show discordant or opposite effects. The data analyzed en masse suggest an inverse relationship between LRO Nile red and lipid mass. (B) LRO Nile red versus blue autofluorescence following RNAi knockdown of 79 genes that affect LRO Nile red indicate that the genetic regulation of LRO Nile red is distinct from the regulation of blue autofluorescence. A group of genes involved in LRO biogenesis and function show concordant decreases in LRO Nile red and blue autofluorescence. *kat-1* shows a high LRO Nile red and high blue autofluorescence. However, most gene inactivations show discordant Nile red and blue autofluorescence. Red dots indicate labeled genes on the graph to distinguish them from non-labeled genes (black dots).

To examine the broader genetic architecture regulating lysosome-related organelle biology, we re-visited a set of 407 gene inactivations reported to positively or negatively affect Nile red accumulation [16]. We added another 164 genes to this list encoding genes annotated to regulate metabolism in *C. elegans* (Table S1). To determine the most robust regulators of LRO Nile red among this group we examined the Nile red phenotype after gene inactivation of each gene by RNAi in an enhanced RNAi mutant (*eri-1*) using a quantitative microscopy assay for feeding LRO Nile red at day 1 of adulthood. Genes were tested using 6 biological replicates to ensure reproducibility of the observations. This analysis identified 79 gene inactivations that induce quantitatively different LRO Nile red compared to control RNAi, 56 of which were from the original 407 reported LRO Nile red regulatory genes (Figure 6 and Table S1). This was a stringent cutoff, correcting for multiple hypothesis testing. If criteria were loosened to significance at an uncorrected *P*<0.05, a total of 210 genes met the cutoff, comprising 131 of the original 407 genes previously reported affect Nile red staining and 79 annotated metabolic regulators (Table S1).

Nile red, when fed to *C. elegans*, was assumed to be an indicator of fat mass [16], [31], [32] but it is now thought that the LRO Nile red compartment is distinct from neutral lipid storage in *C. elegans* [14], [20]–[23]. However, fixation-based methods of staining lipids do highly correlate with biochemically measured lipid mass [14], [20]. To systematically study how the regulation of LRO Nile red may correlate with changes in *C. elegans* lipid levels, we utilized a validated, fixation-based staining protocol for highlighting fat stores in the worm [33]. We knocked down all 571 genes by RNAi in *eri-1* worms and stained for lipid in biological quadruplicate (Figure 6B and Table S2). Fixative based staining with Nile red unlike vital staining with Nile red reveals true lipid droplets [20], [21], [33]. To avoid confusion, we refer to the fixative Nile red result as lipid droplet staining. This analysis indicated that there is a negative correlation between LRO Nile red and lipid levels in the worm (Figure 6A and Table S2). Few gene inactivations caused correlated changes in LRO Nile red and lipid levels. Among them were *elt-2* and the protein kinase A (PKA) catalytic subunit ortholog *kin-1*.

To determine the systematic relationship between LRO Nile red accumulation and LRO autofluorescence, we assessed the effect of RNAi knockdown of the most robust LRO Nile red regulators with significance scores passing a corrected threshold of P<0.05, or 79 genes, in wild type worms, rather than the *eri-1* enhanced RNAi mutant used in our primary screen, in biological quadruplicate and assessed LRO Nile red and autofluorescence (Figure 6B). Under these conditions, many gene inactivations in wild type caused less pronounced effects on LRO Nile red than inactivations in the *eri-1* background, and some, e.g. *elt-2*, *obr-2*, *hhat-2*, and *daf-9*, had opposite effects in N2 versus *eri-1* worms (Figure S3). However, overall, these results indicate that while a large number of canonical LRO regulatory genes, e.g. *glo-3*, *pgp-2*, and adaptin subunits *apb-3* and *apm-3* cluster together and demonstrate low LRO Nile red and autofluorescence, a large number of genes demonstrate dissociation between LRO Nile red and autofluorescence. Perhaps the most extreme example was inactivation of the GATA transcription factor *elt-2*, which leads to a ∼3 fold increase in LRO Nile red but a ∼4 fold reduction in intestinal autofluorescence (Figure 6B). In contrast, knockdown of the related transcription factor *elt-7* led to a decrease in LRO Nile red and an increase in autofluorescence. Thus there are many examples of separation of LRO Nile red and autofluorescence as it might represent age pigment.

### Gene network regulating LRO autofluorescence and LRO Nile red accumulation

**Figure 7:**
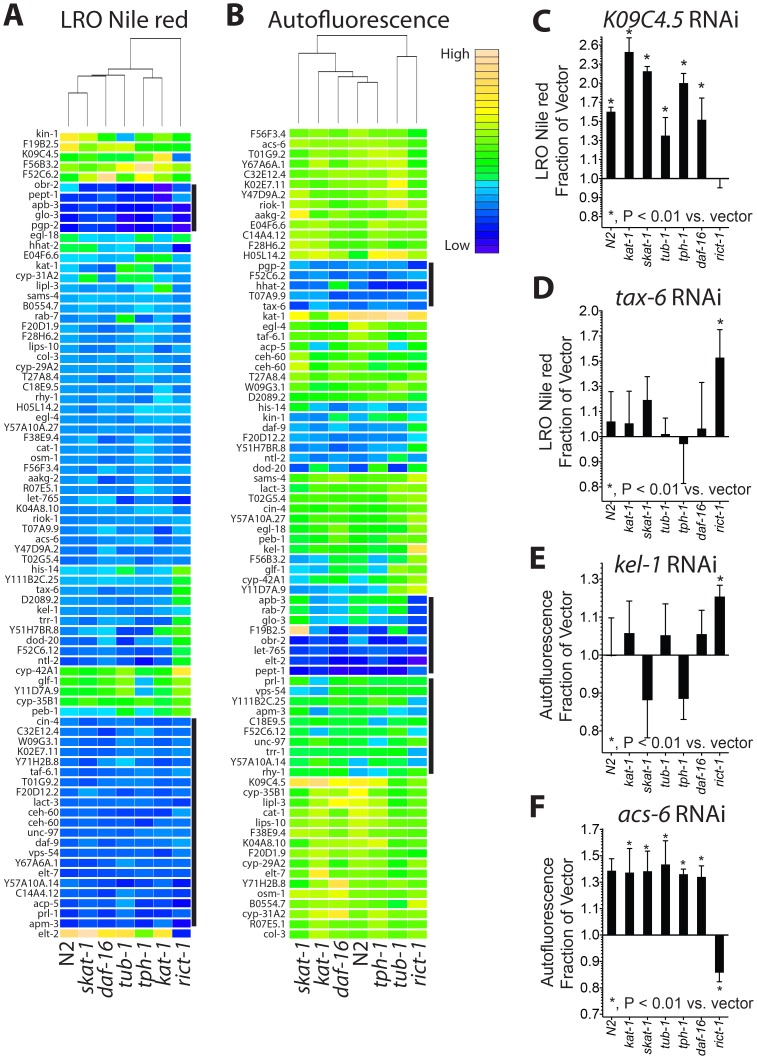
Gene-gene interaction network underlying LRO accumulation of Nile red and autofluorescence. (A) Seventy-nine genes affecting LRO Nile red were inactivated by RNAi in wild type worms and 6 genetic mutants with altered LRO Nile red phenotypes to determine gene-gene interactions. Genes were clustered by *k*-means clustering, with the heatmap indicating genes that increase LRO Nile red in yellow and decrease LRO Nile red in blue. No inference can be made on the absolute magnitude of the effect from the heatmap as data from each mutant for all 79 genes are normalized and scaled (see methods and table S3 for absolute fold change differences). Genes knocked down by RNAi are along the vertical axis and mutant backgrounds are along the horizontal axis. Two clusters of genes that show decreases in all mutants tested are indicated by the black bars to the immediate right of the heatmap. Mutants are organized by hierarchical clustering, indicating that overall *kat-1* and *tph-1* which lacks serotonin cluster most closely to each other (dendrogram on top of the heatmap). (B) The same 79 genes were inactivated in the same seven genetic backgrounds, imaged for blue autofluorescence and organized by cluster analysis as in A. Three clusters of genes that have known regulators of LRO biogenesis are indicated by the black bars along the vertical axis to the right of the heatmap (see also table S4). (C) The solute transporter *K09C4.5* RNAi increases LRO Nile red in all strains tested except the TOR complex 2 mutant *rict-1*. (D) RNAi to the catalytic subunit of the protein phosphatase calcineurin, *tax-6*, led to a decrease in LRO Nile red in all strains tested except *rict-1* in which there was a small but measurable increase. (E) RNAi to the Kelch domain protein *kel-1* increases blue autofluorescence only in a *rict-1* mutant. (F) RNAi to acyl-coenzyme A synthetase *acs-6* increases blue autofluorescence in a manner genetically dependent upon TOR complex 2 mutant *rict-1*. (C–F, N as in table S3 and S4; significance by unequal variance, two-tailed Student's t-test with Bonferroni step-down correction.)

The 79 genes that significantly modulate LRO Nile red when knocked down by RNAi in *eri-1* worms were also knocked down by RNAi in wild-type *C. elegans* (N2 Bristol) and a set of 6 genetic backgrounds with altered lysosome-related organelle phenotypes: *kat-1*, *skat-1*, *tub-1*, *tph-1*, *daf-16*, and *rict-1*. LRO Nile red and autofluorescence were examined (Figure 7). Data were analyzed by *k*-means clustering, with the number of clusters determined by least squares analysis (Figure S4). Immediately a set of known regulators which are involved in LRO biogenesis were apparent in 2 separate clusters as they reduced LRO Nile red irrespective of genetic background: *glo-3*, *pgp-2*, and adaptin subunits *apb-3*, *apm-3* [8], [13]. Among these were also genes not previously implicated in LRO biogenesis, including the oxysterol binding protein *obr-2* and the dipeptide transporter *pept-1*, as well as vacuolar trafficking gene *vps-54*, acid-phosphatase *acp-5*, protein tyrosine phosphatase *prl-1*, the LIM-domain containing adherens junction adaptor protein *unc-97*, the homeobox transcription factor *ceh-60*, transcription initiation factor *taf-6.1*, beta-lactamase *lact-3*, and steroid hydroxylase *daf-9* (Figure 7A and Table S3). Comparing the effects of these RNAi gene inactivations on autofluorescence, a concordant effect on LRO Nile red and autofluorescence is seen in *pgp-2*, *apb-3*, *apm-3*, *glo-3*, *obr-2*, *vps-54*, *prl-1*, *pept-1*, *unc-97*, and *Y57A10A.14* (Figure 7B and Table S4). These can now be studied for their role in LRO biology.

### Solute transporter *K09C4.5*, *tax-6*, *kel-1*, and *acs-6* form a gene network regulating the LRO in a TOR complex 2-dependent manner

We examined the cluster data for genes that affected LRO biology in a manner dependent upon the evolutionarily conserved heteromeric kinase target of rapamycin (TOR) complex 2. Nonsense mutations in gene encoding the essential TOR complex 2 subunit Rictor (*C. elegans rict-1*) lead to shortened lifespan, high fat mass, elevated LRO Nile red and high levels of age-associated autofluorescence [30], [34]. We found one gene inactivation, of the predicted solute transporter *K09C4.5*, that increased LRO Nile red in all strains examined except for the TOR complex 2 mutant *rict-1*, indicating that *rict-1* is genetically downstream of *K09C4.5* regulating LRO Nile red and LRO biology (Figure 7, A and C). Conversely, RNAi to the catalytic subunit of protein phosphatase 2C, *tax-6*, increases LRO Nile red synergistically only in *rict-1* mutants (Figure 7, A and E). These effects are limited to LRO Nile red as a co-regulated change in autofluorescence was not seen. There are additional genes in a cluster with *tax-6* that show similar biology and can now be studied with regard to their interaction with *rict-1* (full dataset in Table S3).

Alternatively, we found many unique interactions of TOR complex 2 mutant *rict-1* with the 79 LRO genes when aging-related autofluorescence was investigated. Autofluorescence is specifically increased by *kel-1* RNAi only in TOR complex 2 mutants (Figure 7, B and E). *kel-1* is a member of the kelch-family group of genes, and regulates pharyngeal development and likely affects feeding [35]. Given that *kel-1* is expressed in the pharyngeal gland and *rict-1* acts to regulate aging in the intestine in a cell-autonomous manner [30], it is likely that these genes act in parallel pathways regulating autofluorescence. Given the synergistic increase only seen in *rict-1* animals, it is possible that *kel-1* is part of a compensatory pathway that protects against premature aging in *rict-1* mutants. We see another cluster of genes exemplified by the acyl-coenzyme A synthetase gene *acs-6* that lead to increases in aging-related autofluorescence in a manner dependent upon *rict-1* (Figure 7, B and F). As autofluorescence accumulates with age, this may be a group of genes that depends upon TOR complex 2 to produce accelerated aging phenotypes (full dataset in Table S4). Similarly, loss of function of *acs-3* has previously been shown to have an impact on LRO Nile red [36].

## Discussion

Lysosome-related organelles have specific functions and share many common features with canonical lysosomes. Disrupted lysosome related organelle biology leads to human Chediak-Higashi and Hermansky-Pudlak Syndromes [3]. In mice, mutations in LRO biogenesis genes leads to alterations in coat color. In *C. elegans*, LROs are responsive to aging as they are the site of age-associated increases in autofluorescence. LROs are also the site of concentration of the vital dye Nile red when fed to the worm. We identified that serotonin regulates LRO biology in a manner that is disrupted in a mutant for the ketothiolase *kat-1*. By orthology, *kat-1* is predicted to catalyze the final step in mitochondrial beta-oxidation of fatty acids, but could also catalyze the reverse reaction, generating the ketone body acetoacetate from two acetyl-Coenzyme A molecules [18]. By sequence homology and KEGG pathway examination, *kat-1* might also play a role in terminal oxidation of hydrophobic or branched chain amino acids such as tryptophan or isoleucine. We have previously found that *kat-1* does not have an altered amount of triglycerides as measured either biochemically with gas chromatography mass spectrometry (GCMS) or by fixative-based lipid staining [14]. Therefore *kat-1* is unlikely to act *via* fundamental alteration of lipid stores in the worm. This could be because *kat-1* plays a larger role in ketone or amino acid metabolism or that one of its two predicted paralogs T02G5.7 or T02G5.4 serves a functionally redundant role. Loss of function mutations in *kat-1* produce hallmarks of premature aging [29] including shortened lifespan, decreased stress resistance, decrease locomotory ability with aging, and evidence of early tissue aging [29]. We postulate that in a *kat-1* mutant, disruption of pathways of beta oxidation and possibly in terminal oxidation of branched chain amino acids leads to increased formation of LRO autofluorescent pigment and increased accumulation of Nile red in the LRO. Given that lipid mass is not abnormal in *kat-1* mutants, the molecular means by which *kat-1* regulates aging related phenotypes remains to be demonstrated.

In *kat-1* animals the elevated LRO Nile red phenotype could be fully suppressed by loss of function in the gene *skat-1*. *skat-1* encodes a predicted 9-transmembrane domain protein targeted either to the LRO and to additional cytoplasmic structures of unclear identity. *skat-1* acts to suppress LRO Nile red entirely *via* expression in the intestine, so we conclude that the increased Nile red uptake into LRO defect induced in a *kat-1* mutant is disabled by the *skat-1* mutation. Much like Nile red, mutation of *skat-1* also fully suppresses the high blue autofluorescence evident in a *kat-1* mutant, but markedly increases the green autofluorescence. One model for this interaction is that the SKAT-1 transmembrane protein in the intestine directly modulates the pH of the LRO. This is not unreasonable given that SKAT-1 localizes to the LRO and is orthologous to proton-coupled amino acid transporters. We suggest that by altering the pH of the LRO, that SKAT-1 prevents accumulation of Nile red, and shifts the emission peak of intestinal autofluorescent material from blue to green.

The nature of the autofluorescent substance in *C. elegans* that accumulates with age was previously thought to be lipofuscin, or oxidation products of cellular proteins and lipids resistant to canonical degradation pathways [7], [10]. Recent findings suggest that rather than lipofuscin, that the autofluorescent substance in *C. elegans* LROs is anthranilate, a breakdown product of tryptophan [37]. In *Drosophila*, kynurenine synthesis as a product of tryptophan catabolism produces autofluorescent globules in the fat body [38]. A role for tryptophan catabolism in the accumulation of intestinal blue autofluorescence has been suggested [39], and, decreased L-kynureninase activity is associated with a shift in autofluorescent material from the blue to the green spectrum [40]. As tryptophan may be metabolized in part in a *kat-1* dependent manner, it is possible that accumulation of kynurenine, anthranilate, or their metabolites is responsible for the high LRO blue autofluorescence in a *kat-1* mutant. This possibility remains to be investigated. Consistent with the notion that mutation of *skat-1* affects LRO pH, tryptophan metabolites are known to undergo shifts in fluorescence excitation and emission at different pH values [41]. Given that serotonin is a tryptophan derivative, it is possible that the mode of serotonin action in the LRO to decrease LRO Nile red is through alteration of tryptophan catabolism in the intestine. This could explain why ambient levels of serotonin have little effect on LRO Nile red but rather elevated exogenous levels or those seen in ciliary mutants, e.g. *tub-1* are needed to evoke a change in LRO Nile red accumulation.

In many assays LRO Nile red and intestinal autofluorescence are correlated. Strains that are short-lived (e.g. *daf-16*, *rict-1*, *kat-1*) have elevated levels of autofluorescence and LRO Nile red, and strains that are long-lived (e.g. *daf-2*, *daf-7*, *eat-2*) tend to have lower levels of both [14], [30]. Many mutants with altered LRO biology have shortened or extended lifespan and stress resistance. For example, in long-lived *daf-2* animals, there is less LRO accumulation of autofluorescence and Nile red; conversely in short-lived *daf-16* animals, there is accelerated accumulation of LRO Nile red and autofluorescence [14]. However, it is not true that all long-lived mutants show decreased LRO Nile red and autofluorescence and that short lived mutants show increased LRO Nile red and autofluorescence [42].

We identified some of the gene networks that are responsible for regulating LRO Nile red and aging-associated autofluorescence. In some genetic backgrounds LRO Nile red and autofluorescence agree, for example high levels in *kat-1* mutants and low levels in gene inactivations that disrupt LRO biogenesis or function (*pgp-2*, *glo-3*, *apm-3*, and *apb-3*). However, we identified many gene inactivations that cause opposite LRO Nile red and autofluorescence phenotypes, indicating that these phenomena do not highlight identical biology and are not simply parallel indicators of aging. We have also found that in many genetic backgrounds, LRO Nile red does not correlate with fixation-based staining of lipids. This confirms that when fed to living *C. elegans* at conditions used in this study and previously published [16], Nile red fluorescence does not highlight major lipid stores. Finally, by systematically studying examining gene-gene interactions we have identified pathways that regulate LRO biology in a manner dependent upon the conserved kinase complex TOR complex 2. The results presented can also shed light on the genetic mechanisms underlying aging related changes in the biology of the LRO and its accumulation of autofluorescent pigments.

Given the many examples of separation of these two phenotypes in our large-scale RNAi study presented here, we conclude that LRO Nile red and autofluorescence likely represent distinct biologies and can be mechanistically separated. Our data indicate that alteration in LRO function through mutation of *skat-1* can lead to reduced LRO Nile red and a green shift in intestinal autofluorescence. The data in aggregate illustrate that although both phenotypes are centered around the LRO, different biologies likely contribute to the accumulation of autofluorescence and Nile red. We show that LRO Nile red is differently regulated by RNAi of several genes in wild type versus enhanced RNAi mutant *eri-1*. The disparate regulation of LRO Nile red in wild type versus *eri-1* represents one of two possibilities, either an effect that is dependent upon increased penetrance of RNAi into normally RNAi resistant tissues in the *eri-1* mutant, e.g. the nervous system, or increased efficiency of RNAi knockdown in RNAi sensitive tissues. Excitingly, the data suggest differential roles for LRO regulatory genes in different tissues, and illuminate potential future directions for investigation of non-cell autonomous modes of regulation of LRO biology.

By systematic examination of a set of 79 genes that affect LRO Nile red, we have identified a number of phenotypic clusters of LRO regulatory genes. These genes, like *skat-1*, may identify components in the sorting of Nile red to the LRO or the sorting of endogenous lipids and other macromolecules such as the autofluorescent gut molecules to the LRO. The clusters of particular gene interactions may correspond to modules of biological function in the LRO. We find that there are many gene inactivations that affect Nile red sorting to the LRO but not sorting of endogenous autofluorescent molecules, and vice versa. There are other gene inactivations that disrupt both processes. Thus while in some instances, for example in the case of genes involved in the biogenesis of the LRO and for *kat-1* mutants, LRO Nile red and autofluorescence are concordant, there are many instances where discordant effects are seen. We also find that LRO Nile red is not concordant with changes in fat mass as determined by fixation-based lipid staining. If anything the data suggest an anticorrelation with LRO Nile red and fat mass. And we found that LRO biology is strongly dependent upon the genetic background examined. We found multiple instances where changes in autofluorescence or LRO Nile red were specific to a given genetic background. Specifically, we found effects of inactivation of the solute transporter *K09C4.5*, calcineurin phosphatase catalytic subunit *tax-6* and the Kelch domain protein *kel-1* to be dependent upon the function of TOR complex 2. As the LRO, like the lysosome, plays a key role in trafficking of proteins and degradation products around the cell, it is likely that exploration of these genetic interactions will inform our knowledge of processes such as autophagy, protein synthesis and turnover, and potentially inform human diseases of lysosomes or lysosome related organelles.

## Materials and Methods

### Strains used

N2 Bristol was used as the wild type strain. The following mutant strains were used: GR1373 *eri-1*(*mg366*), MGH55 *daf-16*(*mgDf47*), MGH112 *tph-1*(*n4622*), MGH102 *kat-1(mg449)*, MGH104 *skat-1(mg459)*, MGH54 *tub-1(nr2004)*, MGH53 *kat-1(mg368) tub-1(nr2004)*, LC35 *cat-4(ok342)*, CB1112 *cat-2(e1112)*, MGH1 *rict-1*(*mg451*), For tissue distribution of *skat-1*, MGH273 *alxEx50*: *Ex*[*F59B2.5p*::mRFP *myo-2p*::GFP]. 1.4 kb of upstream *F59B2.5* promoter sequence was used. For intestinal *skat-1* rescue experiments, MGH129 *kat-1(mg449);skat-1(mg459);alxEx6*[*vha-6p*::*skat-1*::GFP *myo-2p*::mCherry], *and* MGH130 *kat-1(mg449);skat-1(mg459);alxEx7*[*vha-6p*::*skat-1*::GFP *myo-2p*::mCherry] with 0.8 kb of upstream *vha-6* promoter; for neuronal *skat-1* rescue, MGH133 *kat-1(mg449);skat-1(mg459);alxEx10*[*rab-3p*::*skat-1*::GFP *myo-2p*::GFP]and MGH134 *kat-1(mg449);skat-1(mg459);alxEx11*[*rab-3p*::*skat-1*::GFP *myo-2p*::GFP] 2.2 kb of upstream *rab-3* promoter was used.

### Vital dye assays for lysosome related organelle Nile red, LysoTracker Red, and C1-BODIPY-C12

The feeding Nile red assay for LRO Nile red was conducted by seeding wild-type or mutant *C. elegans* on NGM plates containing either *E. coli* OP50 or HT115 supplemented with either 50 ng/mL µM Nile red final (diluted fresh into 100 µL M9 media per plate from 500 µg/mL stock in acetone and added to the top of E. coli plates and allowed to dry), 20 ng/mL C1-BODIPY 500/510-C12 final (diluted fresh into 100 µL M9 media per plate from 200 µg/mL stock in DMSO), or 1 µM LysoTracker Red (diluted fresh into 100 µL M9 media from 1 mM stock in DMSO) (all from Invitroen) as L1 following overnight hatching and synchronization at 20°C in minimal media. Imaging and quantitation was conducted after growth at 20°C as day 1 adults using a Zeiss Axioplan microscope and Axiovision software (Figures 1–4), or a Leica DM6000 microscope and MMAF software (Figures 5–7). For Figures 1–5, at least 30 animals were imaged on at least 2 separate occasions, and results were consistent between experiments. For figures 6 and 7, wild type or mutant animals treated with RNAi were imaged in 96-well format in biological quadruplicate or 6× replicates as indicated in the text. All LRO Nile red analyses were carried out on animals grown at 20°C.

### Autofluorescence assay

Autofluorescence in day 1 adult worms was quantified by after growth at 20°C by picking worms from plates into M9 buffer containing levamisole as a paralytic, and mounting animals in multiwell Teflon-masked microscope slides. Images were acquired with a Leica DM6000 microscope outfitted with a standard DAPI filter set (for blue spectrum autofluorescence) or GFP filter set (for green spectrum autofluorescence) and MMAF software (Figure 5). Identical exposure times were used for each set of animals imaged within an experiment. At least 30 animals were imaged on at least 2 separate occasions, and results were consistent between experiments.

### Serotonin treatment of *C. elegans* and isolation of serotonin resistant mutants

Synchronous populations of wild type or mutant worms at the L1 stage were dropped on to NGM plates containing *E. coli* bacteria. After 36 hours at 20°C, serotonin at the concentrations indicated was added to the top of the bacterial lawn in M9 minimal media, allowed to dry in the dark in a laminar flow hood, and worm plates were returned to the 20°C incubator. Worms were imaged after an additional 48 or 72 hours, and 48 hours was chosen as the time point for further study based upon the effect on LRO Nile red being maximal. For isolation of serotonin resistant mutants, 120,000 haploid genomes were screened in the F2 generation by EMS mutagenesis. Synchronous F2 animals at the L1 stage were dropped on to *E. coli* OP50 lawns containing 1 µM Nile red on 10 cm NGM plates and grown at 20°C for 36 hours. Thereafter serotonin to a final concentration of 2.5 mM in the agar was added to the top of the bacterial lawn containing worms, allowed to dry, and incubated for an additional 48 hours prior to screening for individual worms with elevated residual LRO Nile red staining. Six independent mutants with elevated LRO Nile red staining were mapped using the multiply polymorphic strain CB4856 to mid chromosome II. After narrowing the interval, RNAi, complementation, and direct sequencing were used to identify causal mutations in *kat-1*.

### Quantitative PCR for *kat-1* mRNA

All assays were conducted in biological triplicate on 5000 worms per sample. Wild type *C. elegans* were dropped as synchronous L1 larvae onto NGM agarose plates containing E. coli OP50 and allowed to grow for 36 hours prior to the addition of 2.5 mM serotonin in M9 minimal media or M9 as vehicle. Worms were incubated for an additional 24 or 48 hours on serotonin or vehicle plates prior to harvest for RNA preparation. For mutant analysis, N2, *tph-1* or *tub-1* animals were dropped as synchronous L1 larvae onto NGM agarose plates with OP50 and allowed to grow to the mid-L4 stage prior to harvest for RNA preparation. Serotonin treated or mutant worms were harvested by washing off of plates with M9 media and washed an additional 4 times with an excess of M9 buffer, allowing worms to settle by gravity between washes. Worms were flash frozen in liquid nitrogen until RNA preparation with TRIzol (Invitrogen) per manufacturer instructions. RNA was treated with DNAse free RNAse and reverse transcribed with the Quantitect reverse transcription kit (Qiagen) prior to real-time PCR. Real time PCR was conducted on kat-1 mRNA or snb-1 mRNA (control) using Quantitect SYBR Green PCR reagent (Qiagen) according to manufacturer instructions, and fold change of kat-1 expressed following normalization to the abundance of snb-1 mRNA by the δδCt method using experimentally determined efficiency values. Primer sequences used were: *kat-1* F primer 5′-tcacctcgctgagaactgttt-3′; *kat-1* R primer 5′-ttcctgtgaggcaacaatga-3′ (product 107 nt); *snb-1* F primer 5′-ccggataagaccatcttgacg-3′; and *snb-1* R primer 5′- gacgacttcatcaacctgagc-3′ (product 121 nt).

### Isolation of *skat-1* (*F59B2.2*) mutant

*C. elegans* N2 Bristol strain was mutagenized with EMS, and the resultant F2 generation was screened for decreased staining with the vital dye Nile Red [18]. *skat-1*(*mg459*) was identified by back-crossing three times to N2 Bristol and positionally cloned based upon polymorphisms between N2 and the multiply polymorphic *C. elegans* strain CB4586. Following narrowing of the genetic interval to ∼300 genes, *skat-1* was cloned by direct sequencing following phenocopy of the very low LRO Nile red phenotype with *skat-1* RNAi in a *kat-1*(*mg449*) mutant.

### Systems level analysis of LRO autofluorescence and LRO Nile red

RNAi clones were isolated from a genome-wide *E. coli* feeding RNAi library and fed to *C. elegans* as previously described [43] in 96-well format using NGM agarose supplemented with 5 mM IPTG (US Biologicals) and 100 µg/mL carbenicillin. Plates also contained 1 µM Nile red, for LRO Nile red analysis or no additional additive for autofluorescent analysis. Synchronous populations of wild type or mutant worms were obtained by bleach treatment of gravid adults [44], dropped onto 96-well RNAi plates as synchronous L1 and at day 1 of adulthood, washed from RNAi plates, paralyzed with 30 mM 2,3-butanedione monoxime (Sigma) or 10 µg/mL L-tetramisole (Sigma), mounted on a 96-well teflon slide (Trevigen) and imaged on a Leica DM6000 microscope in brightfield and fluorescent channels with uniform exposure times (DAPI for autofluorescence, Texas Red filter set for Nile red) at 25× magnification. This magnification was specifically chosen as the depth of field of the objective captures the entire worm in focus. Image analysis was carried out using custom MATLAB (The Mathworks) scripts, parallelized for speed. Full well images were built by tiling raw brightfield images.

#### Well finding

Full well images were built by tiling raw brightfield images. Each full well image was blurred using a (30×30 pixel) Gaussian, scaled to account for its 12 bit dynamic range within a 16 bit image, then converted to 8 bit to facilitate rapid thresholding. The threshold level was determined as L*F where L is the threshold determined using Otsu's method, and F is an empirical factor equal to 0.9. After thresholding, holes are filled and the largest connected region is chosen as the well. Then well is eroded with a (30 pixel diameter) disk structuring element. To avoid edge effects due to partial well images, we first pad the well with a (30 pixel) border before the erosion, then restore the original image size after erosion.

#### Object finding

The well is segmented into three masks characterized as background, junk (foreground objects that should not be quantified), and objects. First, each full well brightfield image is bottom hat filtered, using a (20 pixel diameter) disk structuring element, to give an image with much less variability in background intensity. The background-stabilized image is scaled to produce 1% saturation and then quantized to 8 bits. Otsu's method is then applied to the well region only, to give the Otsu threshold L. If the level L and effectiveness E are adequate, we threshold using the level 1-F*(1-L), where L is the threshold determined using Otsu's method, and F is an empirical factor equal to 0.9. F<1 biases this operation towards decreased false positive rate of object identification at the expense of reduced well area. We also perform image closure (3 pixel disk) to eliminate small holes. We typically trigger a second “outlier” method when the effectiveness E is low (<0.7). In this method we blur the image (3×3 pixel Gaussian filter) and identify outlier pixels as those that have intensities beyond a specified boxplot whisker value (nominally 3, which corresponds to >4.6 standard deviations). Next, we filter to eliminate small objects, with the upper and lower size limits allowed to vary between the “threshold” and “outlier” methods, typically with smaller size limits for the “outlier” method. If the largest connected region is very small, a separate “small object” size filter is used. The “threshold”, “outlier” and “small object” methods thus allow progressive detection of smaller and smaller objects (e.g. small worms) while retaining the ability to reject junk (e.g. debris, eggs). Regions that pass all filtering are classified as objects, whereas regions that pass the initial thresholding or outlier analysis but fail the size filter are classified as junk; regions that fail the initial thresholding or outlier analysis are classified as background. Binary masks are then stored for the objects, junk, and background, with the bitwise OR of these three masks equal to the well mask. The identified regions of interest are also flagged for review based on a number of criteria including low thresholding effectiveness or level, low object count, and high junk to object area. All regions of interest are manually reviewed if flagged and excluded from further analysis if poorly indicative of true worm area.

#### Quantification

Summary statistics are computed based on each of the objects, junk, and background masks including intensity histograms and, as a default, the mean, median and 90th percentile intensity for each mask. Because the background intensity distribution is well approximated by a normal distribution with small standard deviation, we calculate adjusted estimates by subtracting the background mean.

### Systems level analysis of body fat mass using fixation-lipid staining

Fixation based staining with Nile red, which reliably stains neutral lipid droplets, was conducted as previously indicated [33]. In brief, following feeding RNAi in 96-well format as above, animals are washed from RNAi plates, fixed in 40% isopropanol, stained in 3 µg/mL Nile red in 40% isopropanol, washed in PBS with 0.01% Triton-X100, mounted on 96-well Teflon slides and imaged as above for LRO Nile red, except rather than Texas red imaging, GFP filter sets are used. Images are analysed as above.

### Statistical and *k*-means cluster and statistical analysis

Statistical differences between groups were determined using ANOVA, Bonferroni corrected for multiple hypothesis testing. For systems level analysis of LRO Nile red, autofluorescence, and body fat mass, the 90^th^ percentile intensity was used. Differences between RNAi treatments and vector control were determined by unequal variance T test Bonferroni corrected for multiple hypothesis testing. *k*-means cluster analysis was conducted following log_2_ transformation and quantile normalization using R/Bioconductor. Data were visualized using the heatmap.2 function.

## Supporting Information

Figure S1 Analysis of SKAT-1 protein and *skat-1* expression. (A) SKAT-1 homology to yeast, mouse, and human vacuolar, proton-coupled, solute transporters. (B) Predicted membrane topology for 9 transmembrane segments of SKAT-1. (C) Expression of green fluorescent protein driven by the *skat-1* promoter (upstream gene in intron *F59B2.5p*). Head and tail neurons (*top*), intestine and tail neurons (*middle*), ventral nerve cord, intestine, and vulvar muscles (*bottom*) show expression.
(PDF)

Figure S2 LRO accumulation of Nile red, autofluorescent material, and LysoTracker red. (A) Perfect overlap is seen between LRO compartments decorated by PGP-2::GPF, Nile red fed as a vital dye, and intestinal autofluorescence. (B) While most LRO that are decorated by PGP-2::GFP are also positive for LysoTracker red and autofluorescence, we identified distinct populations of intestinal PGP-2 positive granules that did not stain with Lysotracker red (C, *arrows*).
(PDF)

Figure S3 LRO accumulation of Nile red in *eri-1* versus wild type (N2) *C. elegans*. Seventy-nine genes affecting LRO Nile red were inactivated by RNAi in wild type worms (N2) versus eri-1, shown plotted on a logarithmic scale with diagonal equal to unity.
(PDF)

Figure S4 Least squares and principal component analysis of *k*-means clustered data from Figure 7. Least squares analysis of data for 79 genes affecting LRO Nile red indicate a local minimum at 10 clusters for LRO Nile red (A) and plateau at 11 clusters for autofluorescence (C). Principle component analysis of clusters from Nile red (B) and autofluorescence (D) *k* means clusters indicating good separation between clusters. Identity of genes within each cluster is indicated in Table S3 for Nile red and Table S4 for autofluorescence.
(PDF)

Table S1 LRO Nile red in *eri-1* mutants treated with RNAi to 407 genes previously annotated to have feeding Nile red phenotypes and 164 metabolic genes.
(XLSX)

Table S2 Lipid droplet staining in *eri-1* mutants treated with RNAi to 407 genes previously annotated to have feeding Nile red phenotypes and 164 metabolic genes.
(XLSX)

Table S3 LRO Nile red in wild type and 6 genetic mutants with altered LRO Nile red treated with top 79 RNAi affecting LRO Nile red.
(XLSX)

Table S4 Autofluorescence in wild type and 6 genetic mutants with altered LRO biology treated with top 79 RNAi affecting LRO Nile red.
(XLSX)

## References

[1] Kurz T, Terman A, Gustafsson B, Brunk UT (2008) Lysosomes and oxidative stress in aging and apoptosis. Biochim Biophys Acta 1780: 1291–1303. 18255041 10.1016/j.bbagen.2008.01.009

[2] Terman A, Gustafsson B, Brunk UT (2007) Autophagy, organelles and ageing. J Pathol 211: 134–143. 17200947 10.1002/path.2094

[3] Huizing M, Helip-Wooley A, Westbroek W, Gunay-Aygun M, Gahl WA (2008) Disorders of lysosome-related organelle biogenesis: clinical and molecular genetics. Annu Rev Genomics Hum Genet 9: 359–386. 18544035 10.1146/annurev.genom.9.081307.164303 PMC2755194

[4] Kornfeld S, Mellman I (1989) The biogenesis of lysosomes. Annu Rev Cell Biol 5: 483–525. 2557062 10.1146/annurev.cb.05.110189.002411

[5] Mullins C, Bonifacino JS (2001) The molecular machinery for lysosome biogenesis. Bioessays 23: 333–343. 11268039 10.1002/bies.1048

[6] Luzio JP, Poupon V, Lindsay MR, Mullock BM, Piper RC, et al (2003) Membrane dynamics and the biogenesis of lysosomes. Mol Membr Biol 20: 141–154. 12851071 10.1080/0968768031000089546

[7] Brunk UT, Terman A (2002) Lipofuscin: mechanisms of age-related accumulation and influence on cell function. Free Radic Biol Med 33: 611–619. 12208347 10.1016/s0891-5849(02)00959-0

[8] Hermann GJ, Schroeder LK, Hieb CA, Kershner AM, Rabbitts BM, et al (2005) Genetic analysis of lysosomal trafficking in Caenorhabditis elegans. Mol Biol Cell 16: 3273–3288. 15843430 10.1091/mbc.E05-01-0060 PMC1165410

[9] Dell'Angelica EC, Mullins C, Caplan S, Bonifacino JS (2000) Lysosome-related organelles. FASEB J 14: 1265–1278. 10877819 10.1096/fj.14.10.1265

[10] Gerstbrein B, Stamatas G, Kollias N, Driscoll M (2005) In vivo spectrofluorimetry reveals endogenous biomarkers that report healthspan and dietary restriction in Caenorhabditis elegans. Aging Cell 4: 127–137. 15924569 10.1111/j.1474-9726.2005.00153.x

[11] Roh HC, Collier S, Guthrie J, Robertson JD, Kornfeld K (2012) Lysosome-related organelles in intestinal cells are a zinc storage site in C. elegans. Cell Metab 15: 88–99. 22225878 10.1016/j.cmet.2011.12.003 PMC4026189

[12] Rabbitts BM, Ciotti MK, Miller NE, Kramer M, Lawrenson AL, et al (2008) glo-3, a novel Caenorhabditis elegans gene, is required for lysosome-related organelle biogenesis. Genetics 180: 857–871. 18780725 10.1534/genetics.108.093534 PMC2567386

[13] Schroeder LK, Kremer S, Kramer MJ, Currie E, Kwan E, et al (2007) Function of the Caenorhabditis elegans ABC transporter PGP-2 in the biogenesis of a lysosome-related fat storage organelle. Mol Biol Cell 18: 995–1008. 17202409 10.1091/mbc.E06-08-0685 PMC1805080

[14] O'Rourke EJ, Soukas AA, Carr CE, Ruvkun G (2009) C. elegans major fats are stored in vesicles distinct from lysosome-related organelles. Cell Metab 10: 430–435. 19883620 10.1016/j.cmet.2009.10.002 PMC2921818

[15] Mukhopadhyay A, Deplancke B, Walhout AJ, Tissenbaum HA (2005) C. elegans tubby regulates life span and fat storage by two independent mechanisms. Cell Metab 2: 35–42. 16054097 10.1016/j.cmet.2005.06.004

[16] Ashrafi K, Chang FY, Watts JL, Fraser AG, Kamath RS, et al (2003) Genome-wide RNAi analysis of Caenorhabditis elegans fat regulatory genes. Nature 421: 268–272. 12529643 10.1038/nature01279

[17] Srinivasan S, Sadegh L, Elle IC, Christensen AG, Faergeman NJ, et al (2008) Serotonin regulates C. elegans fat and feeding through independent molecular mechanisms. Cell Metab 7: 533–544. 18522834 10.1016/j.cmet.2008.04.012 PMC2495008

[18] Mak HY, Nelson LS, Basson M, Johnson CD, Ruvkun G (2006) Polygenic control of Caenorhabditis elegans fat storage. Nat Genet 38: 363–368. 16462744 10.1038/ng1739

[19] Cohen M, Reale V, Olofsson B, Knights A, Evans P, et al (2009) Coordinated regulation of foraging and metabolism in C. elegans by RFamide neuropeptide signaling. Cell Metab 9: 375–385. 19356718 10.1016/j.cmet.2009.02.003

[20] Brooks KK, Liang B, Watts JL (2009) The influence of bacterial diet on fat storage in C. elegans. PLoS ONE 4: e7545. 19844570 10.1371/journal.pone.0007545 PMC2760100

[21] Yen K, Le TT, Bansal A, Narasimhan SD, Cheng JX, et al (2010) A comparative study of fat storage quantitation in nematode Caenorhabditis elegans using label and label-free methods. PLoS ONE 5: e12810. 20862331 10.1371/journal.pone.0012810 PMC2940797

[22] Zhang SO, Trimble R, Guo F, Mak HY (2010) Lipid droplets as ubiquitous fat storage organelles in C. elegans. BMC Cell Biol 11: 96. 21143850 10.1186/1471-2121-11-96 PMC3004847

[23] Klapper M, Ehmke M, Palgunow D, Bohme M, Matthaus C, et al (2011) Fluorescence-based fixative and vital staining of lipid droplets in Caenorhabditis elegans reveal fat stores using microscopy and flow cytometry approaches. J Lipid Res 52: 1281–1293. 21421847 10.1194/jlr.D011940 PMC3090249

[24] Hobson RJ, Hapiak VM, Xiao H, Buehrer KL, Komuniecki PR, et al (2006) SER-7, a Caenorhabditis elegans 5-HT7-like receptor, is essential for the 5-HT stimulation of pharyngeal pumping and egg laying. Genetics 172: 159–169. 16204223 10.1534/genetics.105.044495 PMC1456143

[25] Song BM, Avery L (2012) Serotonin activates overall feeding by activating two separate neural pathways in Caenorhabditis elegans. J Neurosci 32: 1920–1931. 22323705 10.1523/JNEUROSCI.2064-11.2012 PMC3463504

[26] Horvitz HR, Chalfie M, Trent C, Sulston JE, Evans PD (1982) Serotonin and octopamine in the nematode Caenorhabditis elegans. Science 216: 1012–1014. 6805073 10.1126/science.6805073

[27] Tecott LH, Sun LM, Akana SF, Strack AM, Lowenstein DH, et al (1995) Eating disorder and epilepsy in mice lacking 5-HT2c serotonin receptors. Nature 374: 542–546. 7700379 10.1038/374542a0

[28] Sze JY, Victor M, Loer C, Shi Y, Ruvkun G (2000) Food and metabolic signalling defects in a Caenorhabditis elegans serotonin-synthesis mutant. Nature 403: 560–564. 10676966 10.1038/35000609

[29] Berdichevsky A, Nedelcu S, Boulias K, Bishop NA, Guarente L, et al (2010) 3-Ketoacyl thiolase delays aging of Caenorhabditis elegans and is required for lifespan extension mediated by sir-2.1. Proc Natl Acad Sci U S A 107: 18927–18932. 20956318 10.1073/pnas.1013854107 PMC2973869

[30] Soukas AA, Kane EA, Carr CE, Melo JA, Ruvkun G (2009) Rictor/TORC2 regulates fat metabolism, feeding, growth, and life span in Caenorhabditis elegans. Genes Dev 23: 496–511. 19240135 10.1101/gad.1775409 PMC2648650

[31] Barros AG, Liu J, Lemieux GA, Mullaney BC, Ashrafi K (2012) Analyses of C. elegans fat metabolic pathways. Methods Cell Biol 107: 383–407. 22226531 10.1016/B978-0-12-394620-1.00013-8

[32] Elle IC, Olsen LC, Pultz D, Rodkaer SV, Faergeman NJ (2010) Something worth dyeing for: molecular tools for the dissection of lipid metabolism in Caenorhabditis elegans. FEBS Lett 584: 2183–2193. 20371247 10.1016/j.febslet.2010.03.046

[33] Pino EC, Webster CM, Carr CE, Soukas AA (2013) Biochemical and high throughput microscopic assessment of fat mass in Caenorhabditis elegans. J Vis Exp 73 doi: 10.3791/50180 10.3791/50180 PMC3944676 23568026

[34] Jones KT, Greer ER, Pearce D, Ashrafi K (2009) Rictor/TORC2 regulates Caenorhabditis elegans fat storage, body size, and development through sgk-1. PLoS Biol 7: e60. 19260765 10.1371/journal.pbio.1000060 PMC2650726

[35] Ohmachi M, Sugimoto A, Iino Y, Yamamoto M (1999) kel-1, a novel Kelch-related gene in Caenorhabditis elegans, is expressed in pharyngeal gland cells and is required for the feeding process. Genes Cells 4: 325–337. 10421842 10.1046/j.1365-2443.1999.00264.x

[36] Mullaney BC, Blind RD, Lemieux GA, Perez CL, Elle IC, et al (2010) Regulation of C. elegans fat uptake and storage by acyl-CoA synthase-3 is dependent on NR5A family nuclear hormone receptor nhr-25. Cell Metab 12: 398–410. 20889131 10.1016/j.cmet.2010.08.013 PMC2992884

[37] Coburn C, Allman E, Mahanti P, Benedetto A, Cabreiro F, et al (2013) Anthranilate fluorescence marks a calcium-propagated necrotic wave that promotes organismal death in C. elegans. PLoS Biol 11: e1001613. 23935448 10.1371/journal.pbio.1001613 PMC3720247

[38] Rizki TM (1964) Mutant Genes Regulating the Inducibility of Kynurenine Synthesis. J Cell Biol 21: 203–211. 14153482 10.1083/jcb.21.2.203 PMC2106435

[39] Siddiqui SS, Babu P (1980) Kynurenine hydroxylase mutants of the nematode Caenorhabditis elegans. Mol Gen Genet 179: 21–24. 6935493 10.1007/BF00268441

[40] Bhat SG, Babu P (1980) Mutagen sensitivity of kynureninase mutants of the nematode Caenorhabditis elegans. Mol Gen Genet 180: 635–638. 6936603 10.1007/BF00268072

[41] Fukunaga Y, Katsuragi Y, Izumi T, Sakiyama F (1982) Fluorescence characteristics of kynurenine and N'-formylkynurenine. Their use as reporters of the environment of tryptophan 62 in hen egg-white lysozyme. J Biochem 92: 129–141. 7118867 10.1093/oxfordjournals.jbchem.a133909

[42] Samuelson AV, Carr CE, Ruvkun G (2007) Gene activities that mediate increased life span of C. elegans insulin-like signaling mutants. Genes Dev 21: 2976–2994. 18006689 10.1101/gad.1588907 PMC2049198

[43] Kamath RS, Fraser AG, Dong Y, Poulin G, Durbin R, et al (2003) Systematic functional analysis of the Caenorhabditis elegans genome using RNAi. Nature 421: 231–237. 12529635 10.1038/nature01278

[44] Hope IL, editor (1999) C. elegans: A Practical Approach. New York: Oxford University Press.

