# Supplementary material for: Genetic Regulation of Caenorhabditis elegans Lysosome Related Organelle Function
Source: PLoS Genet. 2013 Oct 24;9(10):e1003908. doi: 10.1371/journal.pgen.1003908 (PMC3812091; doi:10.1371/journal.pgen.1003908)

Figure S1

A

```
ScAVT3   1   [255]GEDLSEEEEEEEETEEEPEE-------EALETESTQLVSREHGRHP-----HKSSTVKAVLLLLKSFVGTGVLFL  318
MmQ8BHK3 1        MSVTKSARSPQVATPLNLDL---PESAKKLQSQDPSPANGSSSESS--KKTKGITGFQTLVHLVKGNMGTGILGL  70
HsSLC36A2 1       MSVTKSTEGPQGAVAIKLDLmspPESAKKLENKDSTFLDESPSESAglKKTKGITVFQALIHLVKGNMGTGILGL  75
CeSKAT-1 1        ---MSEEEGRERAVTVEGDA-------ESMN-DGRALV-QPPARSG-----DVITPTRAVLTLSKSMFNAGCFSL  58

ScAVT3   319 PKAFHNGGWGFSALCLLSCALISYGCFVSLITTKDKV-----------------GVDGYGDMGRILYGPKMKFAILSSIA  381
MmQ8BHK3 71  PLAVKNAGILMGPLSLLVMGLIACHCMHILVRCAQRFCHRLNKPFMDYGDTVMHGLAFSPNAWLQNHAHWGRRVVSFFLI  150
HsSLC36A2 76 PLAVKNAGILMGPLSLLVMGFIACHCMHILVKCAQRFCKRLNKPFMDYGDTVMHGLEANPNAWLQNHAHWGRHIVSFFLI  155
CeSKAT-1 59  PYAWKLGGLWVSFVMSFVIAGLNWYGNHILVRASQHLAKKSDRSALDYGHFAKKVCDYSDIRFLRNNSKAVMYFVNVTIL  138

ScAVT3   382 LSQIGFSAAYTVFTATNLQVFSENFFH-----------LKPGSISLATYIFAQVLIFVPLS-LTRNIAKLSGTALIADLF  449
MmQ8BHK3 151 VTQLGFCCVYIVFLADNLKQVVEAVNSTTISCHKNETVVLTPTMDSRLYMLSFLPVLGLLV-FVRNLRVLTIFSLLANIS  229
HsSLC36A2 156 ITQLGFCCVYIVFLADNLKQVVEAVNSTTNNCYSNETVILTPTMDSRLYMLSFLPFLVLLV-LIRNLRILTIFSMLANIS  234
CeSKAT-1 139 FYQLGMCSVAILFISDNLVNLVGD--H-----------LGGTRHQQMILMATVSLFFILLTnMFTEMRIVSFFALVSSVF  205

ScAVT3   450 ILLGLVYVYVYsiYYIAVNGVASDTMLMFNKADWSLFIGTAIFTFEGIGLLIPIQESMKHPKHFRP---SLSAVMCIVAV  526
MmQ8BHK3 230 MLVSL-VIIAQ--YIIQEIPDASQLPLVASWKTYPLFFGTAIFSFESIGVVLPLENKMKDARGFPT---ILSLGMSIITT  303
HsSLC36A2 235 MLVSL-VIIIQ--YITQEIPDPSRLPLVASWKTYPLFFGTAIFSFESIGVVLPLENKMKNARHFPA---ILSLGMSIVTS  308
CeSKAT-1 206 FVIG-AAVIMQ--YTVQQPNQWDKLPAATNFTGTITMIGMSMYAFEGQTMILPIENKLDNPAAFLApfgVLSTTMIICTA  282

ScAVT3   527 IFISCGLLCYAAFGSDVKTVVLLNFPQdTSYTLTVQLLYALAILLSTPLQLFPAIRILENWTFPSNASGKYNPKVKWLKN  606
MmQ8BHK3 304 LYIAIGALGYLRFGDDIKASITLNLPN-CWLYQSVKLLYVVGILCTYALQFYVPAEII----IPLAVSQVSKRWALPVDL  378
HsSLC36A2 309 LYIGMAALGYLRFGDDIKASISLNLPN-CWLYQSVKLLYIAGILCTYALQFYVPAEII----IPFAISRVSTRWALPLDL  383
CeSKAT-1 283 FMTALGFFGYTGFGDSIAPTITTNVPK-EGLYSTVNVFLMLQSLLGNSIAMYVVYDMFFNGFRRKFGARFPNVPKWLSDK  361

ScAVT3   607 YFRCAIVVLTSILAwVGANDLDKFVSLVGSFACIPLIYIYPPLLHYKASILSGTSRARLLLD----LIVIVFGVAVMAYT  682
MmQ8BHK3 379 SIRLALVCLTCMLA-ILIPRLDLVLSLVGSVSSSALALIIPPLLEVVTYYGEGISPLTVTKD-ALISILGFMGFVVGTYQ  456
HsSLC36A2 384 SIRLVMVCLTCLLA-ILIPRLDLVISLVGSVSGTALALIIPPLLEVTTFYSEGMSPLTIFKD-ALISILGFVGFVVGTYQ  461
CeSKAT-1 362 GFRVFWVLVTYLMA-VLIPKLEIMIPLVGVTSGALCALIFPPFFEMITFWTDWKGLLTYRQRmTKIFINLVVMAIGVFAI  440

ScAVT3   683 SWQT   -IKMWSQ-----------  692
MmQ8BHK3 457 ALDE   LIKSGNSP-ALSNSTMFIQ  478
HsSLC36A2 462 ALDE   LLKSEDSH-PFSNSTTFVR  483
CeSKAT-1 441 IAGV[6]IIQSFSQPdP---------  460
```

B

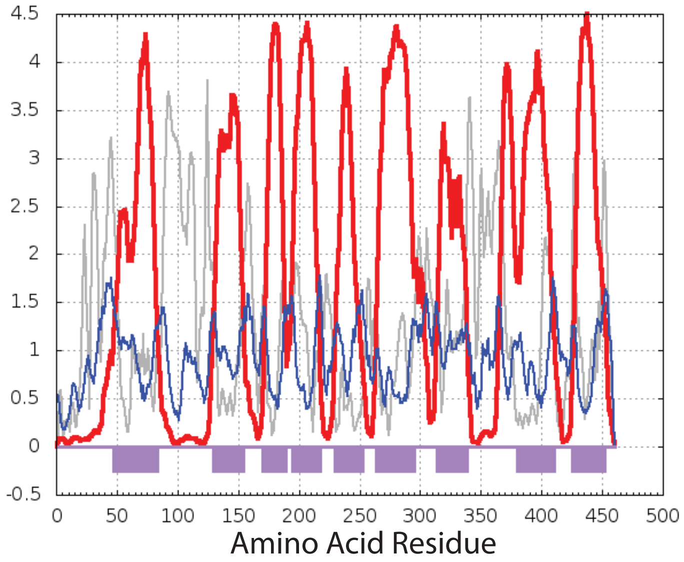

http://split.pmfst.hr/split/4/

Red line: Transmembrane helix preference. (THM index)

Blue line: Beta preference. (BET index)

Gray line: Modified hydrophobic moment index. (INDA index)

Violet boxes (below abscisa): Predicted transmembrane helix position. (DIG index)

C

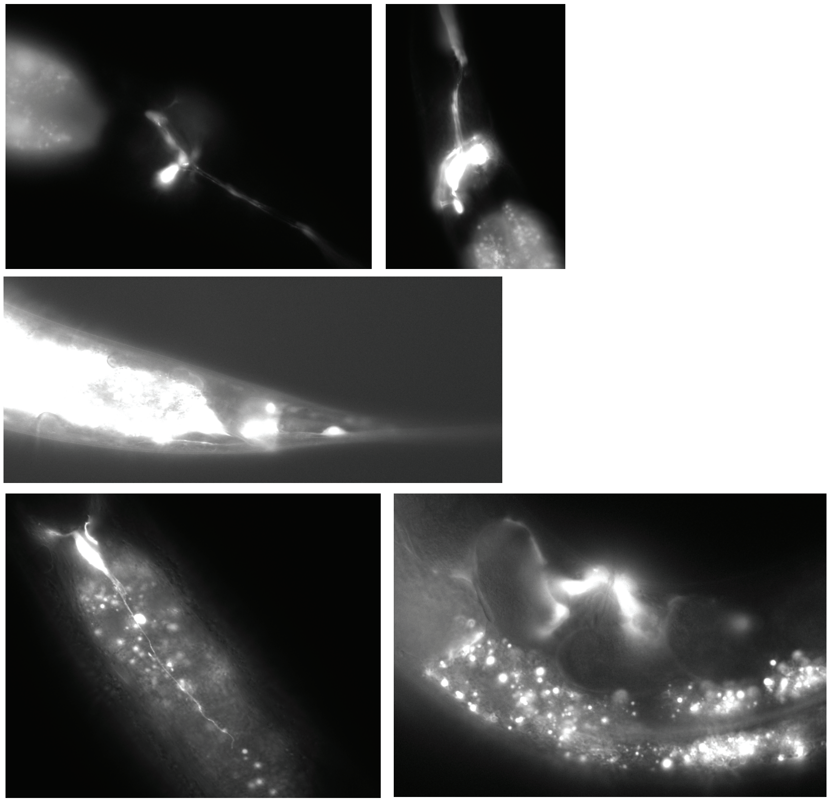

Supplement: Figure S1 — Analysis of SKAT-1 protein and skat-1 expression. (A) SKAT-1 homology to yeast, mouse, and human vacuolar, proton-coupled, solute transporters. (B) Predicted membrane topology for 9 transmembrane segments of SKAT-1. (C) Expression of green fluorescent protein driven by the skat-1 promoter (upstream gene in intron F59B2.5p). Head and tail neurons (top), intestine and tail neurons (middle), ventral nerve cord, intestine, and vulvar muscles (bottom) show expression. (PDF) [file pgen.1003908.s001.pdf]
