# Supplementary figures and images for: Genetic Regulation of Caenorhabditis elegans Lysosome Related Organelle Function
Source: PLoS Genet. 2013 Oct 24;9(10):e1003908. doi: 10.1371/journal.pgen.1003908 (PMC3812091; doi:10.1371/journal.pgen.1003908)

**A**

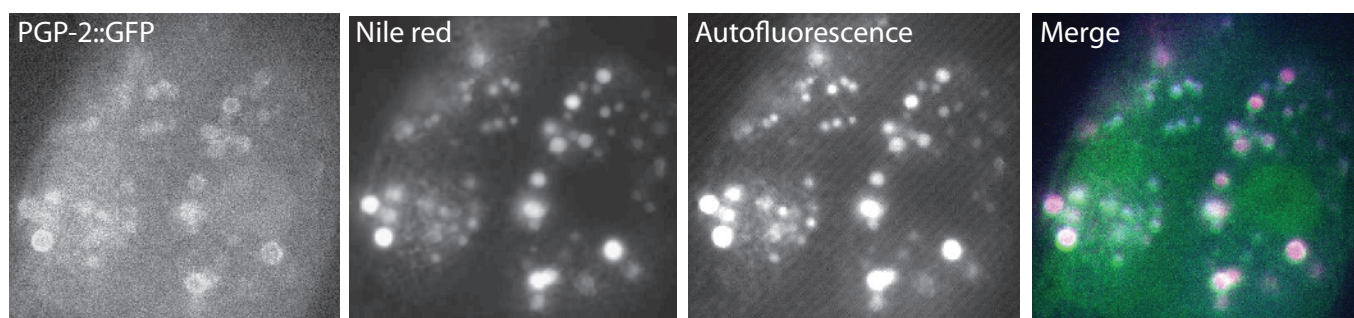

**B**

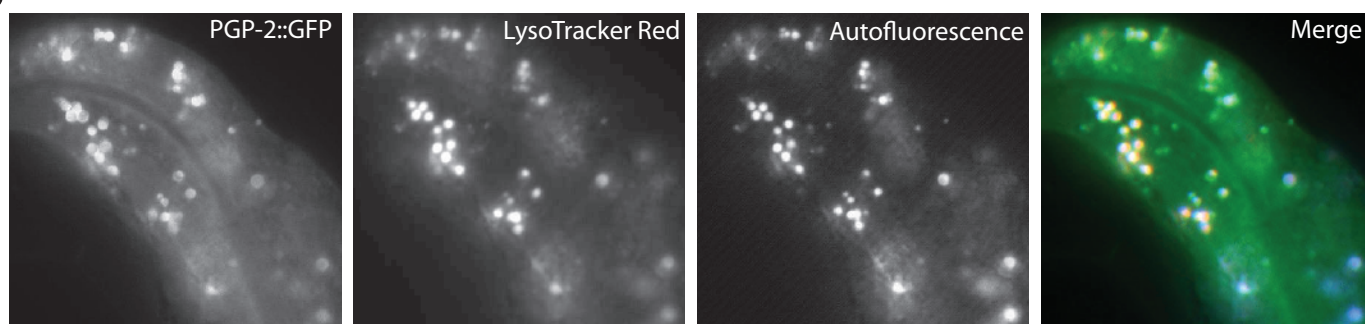

**C**

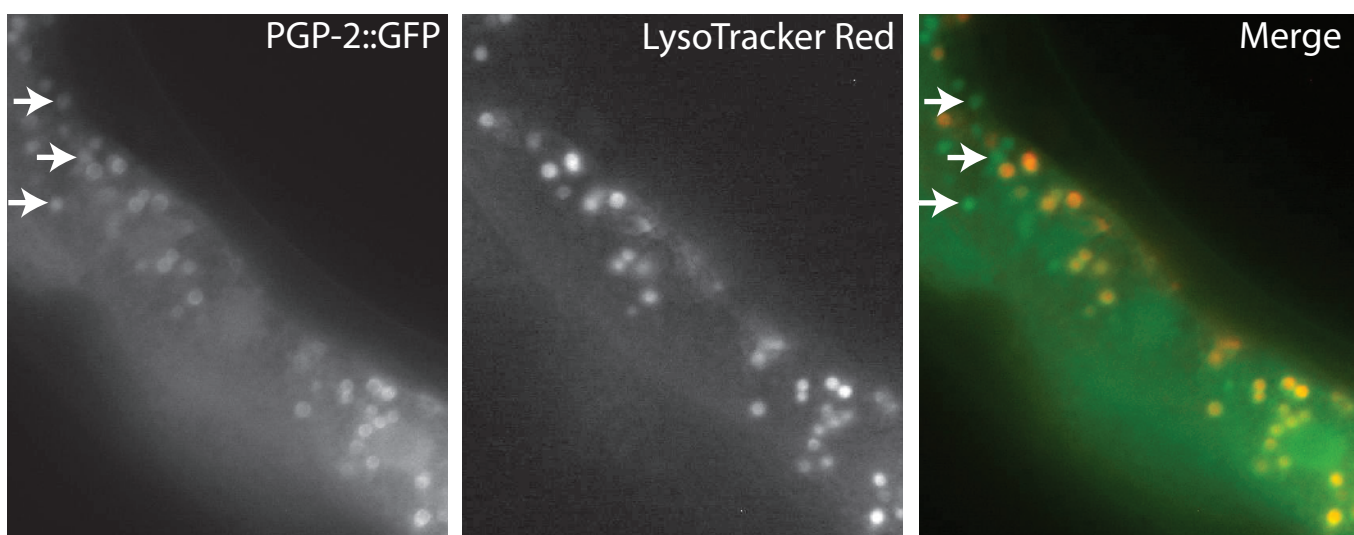

Supplement: Figure S2 — LRO accumulation of Nile red, autofluorescent material, and LysoTracker red. (A) Perfect overlap is seen between LRO compartments decorated by PGP-2::GPF, Nile red fed as a vital dye, and intestinal autofluorescence. (B) While most LRO that are decorated by PGP-2::GFP are also positive for LysoTracker red and autofluorescence, we identified distinct populations of intestinal PGP-2 positive granules that did not stain with Lysotracker red (C, arrows). (PDF) [file pgen.1003908.s002.pdf]

Figure S3

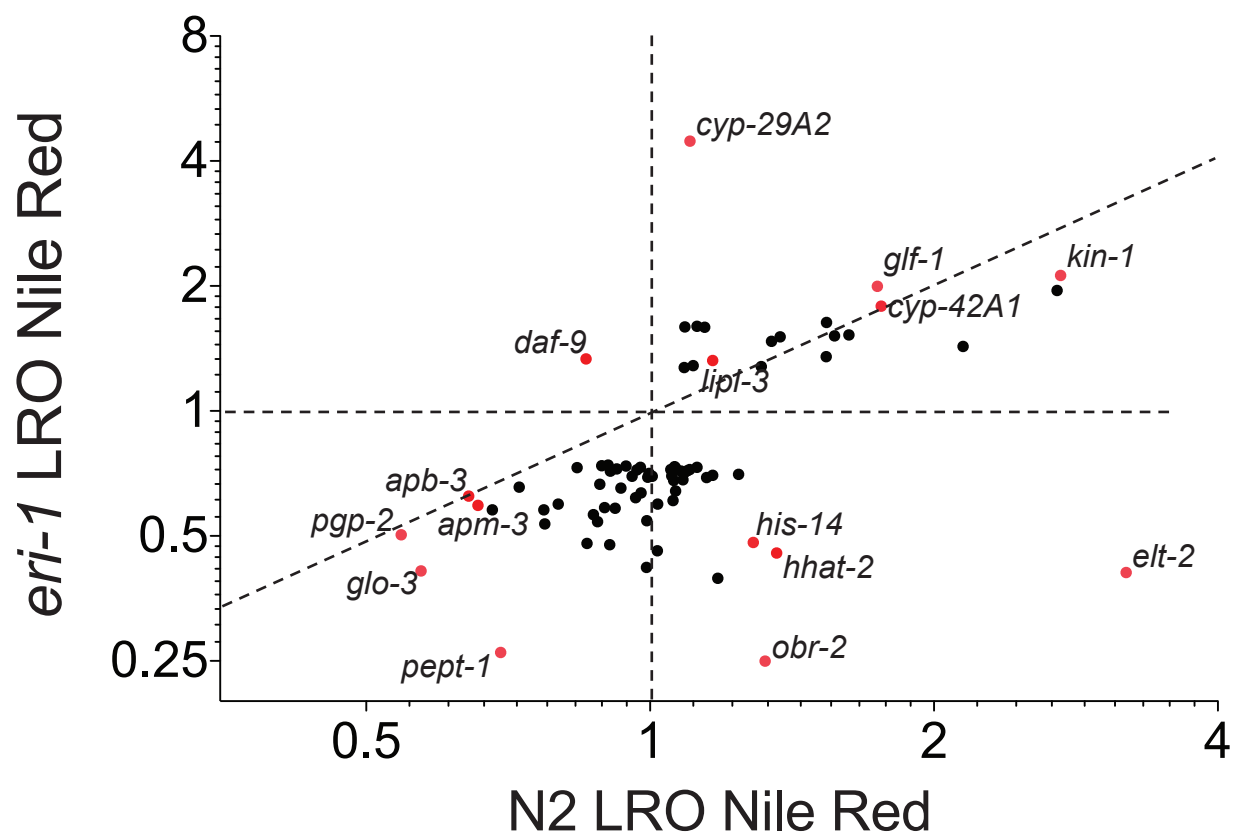

Supplement: Figure S3 — LRO accumulation of Nile red in eri-1 versus wild type (N2) C. elegans. Seventy-nine genes affecting LRO Nile red were inactivated by RNAi in wild type worms (N2) versus eri-1, shown plotted on a logarithmic scale with diagonal equal to unity. (PDF) [file pgen.1003908.s003.pdf]

A

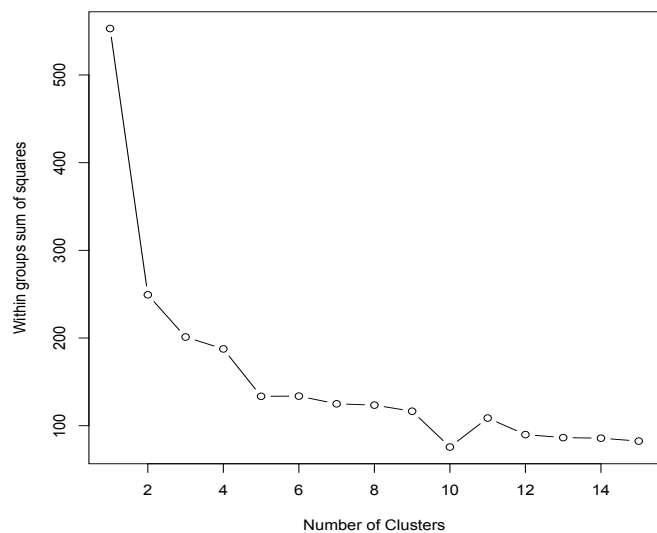

B

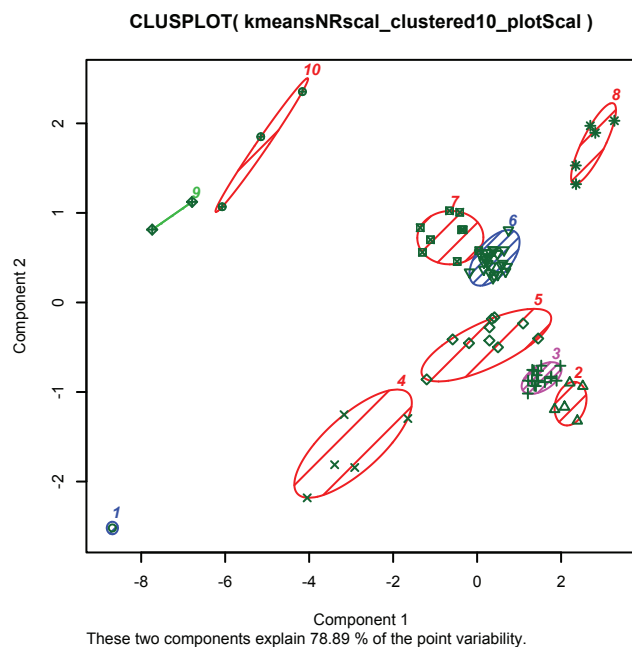

C

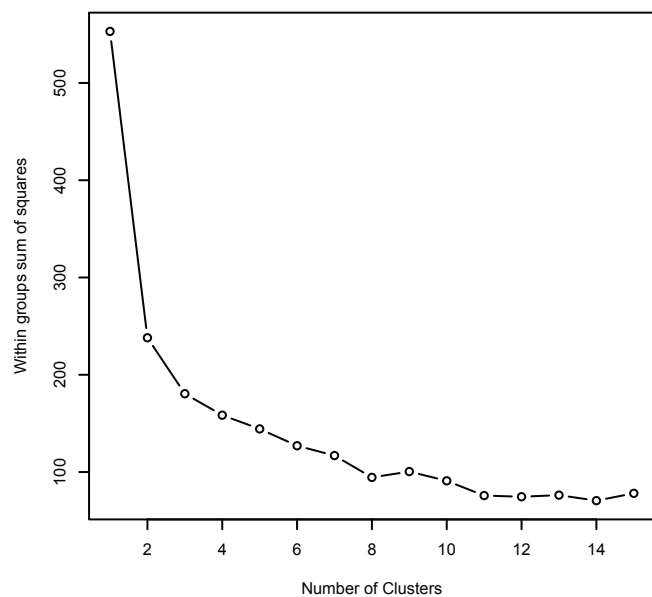

D

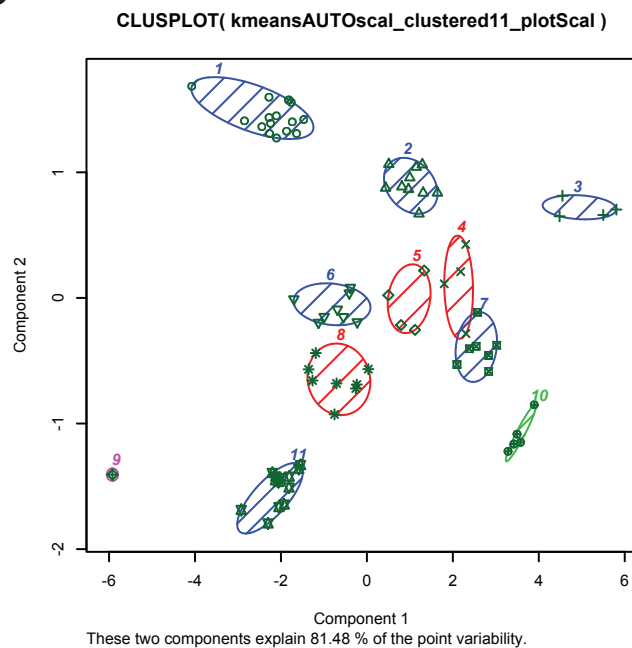

Supplement: Figure S4 — Least squares and principal component analysis of k-means clustered data from Figure 7. Least squares analysis of data for 79 genes affecting LRO Nile red indicate a local minimum at 10 clusters for LRO Nile red (A) and plateau at 11 clusters for autofluorescence (C). Principle component analysis of clusters from Nile red (B) and autofluorescence (D) k means clusters indicating good separation between clusters. Identity of genes within each cluster is indicated in Table S3 for Nile red and Table S4 for autofluorescence. (PDF) [file pgen.1003908.s004.pdf]
